# Nociceptin/orphanin FQ modulates energy homeostasis through inhibition of neurotransmission at VMN SF-1/ARC POMC synapses in a sex- and diet-dependent manner

**DOI:** 10.1186/s13293-019-0220-3

**Published:** 2019-02-12

**Authors:** Jennifer Hernandez, Carolina Fabelo, Lynnea Perez, Clare Moore, Rachel Chang, Edward J. Wagner

**Affiliations:** 1 Department of Basic Medical Sciences, College of Osteopathic Medicine, Western University of Health Sciences, Pomona, CA USA; 2 Graduate College of Biomedical Sciences, Western University of Health Sciences, Pomona, CA USA

**Keywords:** Nociceptin/orphanin FQ, Proopiomelanocortin, Steroidogenic factor-1, Opioid receptor-like 1, Obesity, Insulin resistance

## Abstract

**Background:**

Orphanin FQ (aka nociceptin; N/OFQ) binds to its nociceptin opioid peptide (NOP) receptor expressed in proopiomelanocortin (POMC) neurons within the arcuate nucleus (ARC), a critical anorexigenic component of the hypothalamic energy balance circuitry. It inhibits POMC neurons by modifying neuronal excitability both pre- and postsynaptically. We tested the hypothesis that N/OFQ inhibits neurotransmission at synapses involving steroidogenic factor (SF)-1 neurons in the ventromedial nucleus (VMN) and ARC POMC neurons in a sex- and diet-dependent fashion.

**Methods:**

Electrophysiological recordings were done in intact male and in cycling and ovariectomized female NR5A1-Cre and eGFP-POMC mice. Energy homeostasis was assessed in wildtype animals following intra-ARC injections of N/OFQ or its saline vehicle.

**Results:**

N/OFQ (1 μM) decreased light-evoked excitatory postsynaptic current (leEPSC) amplitude more so in males than in diestrus or proestrus females, which was further accentuated in high-fat diet (HFD)-fed males. N/OFQ elicited a more robust outward current and increase in conductance in males than in diestrus, proestrus, and estrus females. These pleiotropic actions of N/OFQ were abrogated by the NOP receptor antagonist BAN ORL-24 (10 μM). In ovariectomized female eGFP-POMC mice, 17β-estradiol (E_2_; 100 nM) attenuated the N/OFQ-induced postsynaptic response. SF-1 neurons from NR5A1-Cre mice also displayed a robust N/OFQ-induced outward current and increase in conductance that was sexually differentiated and suppressed by E_2_. Finally, intra-ARC injections of N/OFQ increased energy intake and decreased energy expenditure, which was further potentiated by exposure to HFD and diminished by estradiol benzoate (20 μg/kg; s.c.).

**Conclusion:**

These findings show that males are more responsive to the pleiotropic actions of N/OFQ at anorexigenic VMN SF-1/ARC POMC synapses, and this responsiveness can be further enhanced under conditions of diet-induced obesity/insulin resistance.

**Electronic supplementary material:**

The online version of this article (10.1186/s13293-019-0220-3) contains supplementary material, which is available to authorized users.

## Introduction

Nociceptin/orphanin FQ (N/OFQ) is a hepadecapeptide that is similar in structure to the endogenous κ-opioid peptide dynorphin A, yet it does not bind to classical opioid receptors [1, 2]. Its first described physiological role was an increased sensitivity to pain [3], hence the name nociceptin, but it later proved to be involved in many different physiological processes including cardiovascular and gastrointestinal functions, as well as anxiety [4–6].

N/OFQ binds with high affinity to its cognate nociceptin opioid peptide (NOP) receptor, which is a G protein coupled receptor (GPCR) that is structured similar to that of classical opioid receptors like the μ-,δ-, and κ-opioid receptors [1]. It has a widespread distribution within the CNS, with higher quantities in the hypothalamus, hippocampus, amygdala, and brainstem [1, 7]. After agonist activation, NOP receptors trigger different intracellular events including a decrease in the activity of adenylyl cyclase [8]. These receptors also couple directly to G protein-gated, inwardly rectifying K^+^ (GIRK) channels in oocytes [2], as well as neurons in the dorsal raphe [9], locus coeruleus [10], periaqueductal gray [11], and the hypothalamic arcuate nucleus (ARC) [12]. In addition, they negatively modulate both N-type Ca^2+^ channels in SH-SY5Y cells [13] and both N-type as well as P/Q type channels in periaqueductal gray and suprachiasmatic neurons [14, 15].

Bath application of N/OFQ has also been shown to decrease glutamatergic excitatory postsynaptic currents (EPSCs) in rat lateral amygdala, as well as the ARC [16, 17], indicating that N/OFQ acts presynaptically to reduce the amount of glutamate that is released onto its postsynaptic target. Anorexigenic ARC proopiomelanocortin (POMC) neurons have also been shown to be activated from presynaptic glutamatergic inputs emanating from the steroidogenic factor (SF)-1 neurons in the hypothalamic ventromedial nucleus (VMN) [18–21], which have been shown to synapse directly onto POMC neurons [22]. It has also been shown that chemogenetic stimulation of SF-1 neurons causes a decrease in food intake as well as an increase in energy expenditure and that optogenetic stimulation evokes a robust light-induced EPSC in POMC neurons [21]. It has also been known that a lesioning of the VMN causes rampant hyperphagia and obesity [23, 24]. This indicates that this synaptic connection is important in the homeostatic control of energy balance.

As mentioned above, the NOP receptor is expressed within the hypothalamus, where it regulates many homeostatic properties. Within the ARC, it works through GIRK channels to inhibit POMC neurons [12, 25]. When N/OFQ has been administered centrally, it has been shown to increase both food intake and body weight in mice, effects which are more pronounced in high-fat diet (HFD)-fed animals [26]. It also decreases energy expenditure and ultimately leads to hyperleptinemia and hyperinsulinemia [26]. Administration into the ARC has been shown to be particularly efficacious, leading to greater increases in food intake in comparison to other nuclei [27]. Intracerebroventricular (I.C.V) injections of N/OFQ also produce a hypothermic effect in adult rats due to the activation of NOP receptors [28]. Within the VMN, N/OFQ has been shown to hyperpolarize leptin receptor expressing neurons by NOP receptor activation, which can be reversed by the GIRK channel blocker SCH23390 [29]. Injections into the VMN and the nucleus accumbens have also been shown to increase food intake in rats [30]. In addition, co-administration of the selective NOP receptor antagonist [Nphe^1^]NC(1-13)NH_2_ into the third ventricle (3V) reverses the potent orexigenic effects of N/OFQ [31] in male rats, which suggests that N/OFQ regulates energy intake and expenditure, at least in part, by inhibiting neurotransmission at VMN SF-1/ARC POMC synapses.

There is considerable precedence for sex differences in the hypothalamic regulation of energy homeostasis [32]. While we do not know if there are sex differences in the NOP receptor-mediated regulation of energy homeostasis, we do know that there are sex differences in its regulation of nociception. In in vivo electrophysiological recordings taken from the medullary dorsal horn, N/OFQ reduced the *N*-methyl-d-aspartate (NMDA)-evoked responses in males and ovariectomized (OVX) females, but not in proestrus or OVX, estradiol benzoate (EB)-treated females [33]. It has also been shown that an intrathecal injection of N/OFQ attenuated the NMDA receptor-mediated nociceptive response in males and OVX females but not in OVX, EB-treated females [34]. Injection of N/OFQ administered into the same region also produced an increased tail flick latency (TFL) to a thermal stimulus in males as well as OVX females, which was reversed with pretreatment of the NOP antagonist UFP-101. In OVX, EB-treated females, however, there was no significant increase in the TFL upon injection of N/OFQ [34]. It has also been shown that activation of GPR30, estrogen receptor (ER)α, the G_q_-coupled membrane ER (G_q_-mER), but not ERβ abolishes the NOP-mediated antinociception in males and OVX females [35].

We also know that gonadal steroid hormones exert activational effects upon the NOP receptor-mediated regulation of the hypothalamic energy balance circuitry. For example, in ovariectomized, estradiol-primed female rats, the ability of N/OFQ to activate GIRK channels in POMC neurons and decrease miniature EPSC frequency are significantly diminished [36]. Estradiol attenuates these pleiotropic actions of N/OFQ on POMC neurons by binding to either ERα or the G_q_-mER, leading to a signaling cascade that includes PKC, protein kinase A (PKA), phosphatidylinositol-4,5-bisphosphate 3-kinase (PI3K), and PLC [37]. Moreover, progesterone administered to OVX, estrogen-primed females restores the sensitivity of POMC neurons to these pre- and postsynaptic actions of N/OFQ [36].

Thus, there is sufficient precedence to suggest that the NOP receptor-mediated regulation of energy homeostasis and POMC neuronal excitability is sexually differentiated and subject to modulatory influences from diet and gonadal steroid hormones. Moreover, there is compelling evidence that N/OFQ-sensitive glutamatergic input onto POMC neurons arises, in large part, from SF-1 neurons in the dorsomedial VMN [18–20, 22]. We therefore hypothesized that N/OFQ modulates energy homeostasis through an inhibition of excitatory neurotransmission at VMN SF-1/ARC POMC and by modulating postsynaptic conductances in both cell types in a sex- and diet-dependent manner.

## Materials and methods

### Animal models

Adult male and female Topeka guinea pigs (580–879 g; 40–79 days of age) were bred in-house or purchased on demand from Elm Hill Breeding Labs (Clemsford, MA, USA). Male and female NR5A1-Cre mice (18–43 g; 52–144 days of age) were purchased from Jackson Laboratories (Stock #012462) and bred in house. Male and female eGFP-POMC mice (20–43 g; 55–93 days of age) were also purchased from Jackson Laboratories (Stock #009593) and bred in house as well. Animals were housed under a 12:12-h light/dark cycle (light on at 6 a.m. and off at 6 p.m.), with food and water available ad libitum. Intact female NR5A1-Cre and eGFP-POMC mice were checked the day of experimentation by vaginal lavage to evaluate cell cytology and thus determine the stage of the estrous cycle. On the day of experimentation, MRIs were performed using EchoMRI™-100H and EchoMRI™-130 Body Composition Analyzers for Live Small Animals (Mice) and Organs (EchoMRI LLC, Houston, TX, USA) in order to determine the total fat mass as well as lean mass. Fat dissections were also taken from the perirenal, gonadal, and abdominal regions during terminal harvest along with photographic documentation. All procedures were approved by the Western University of Health Sciences’ IACUC and IBC in accordance with institutional guidelines based on NIH standards.

### Diet

**Fig. 1:**
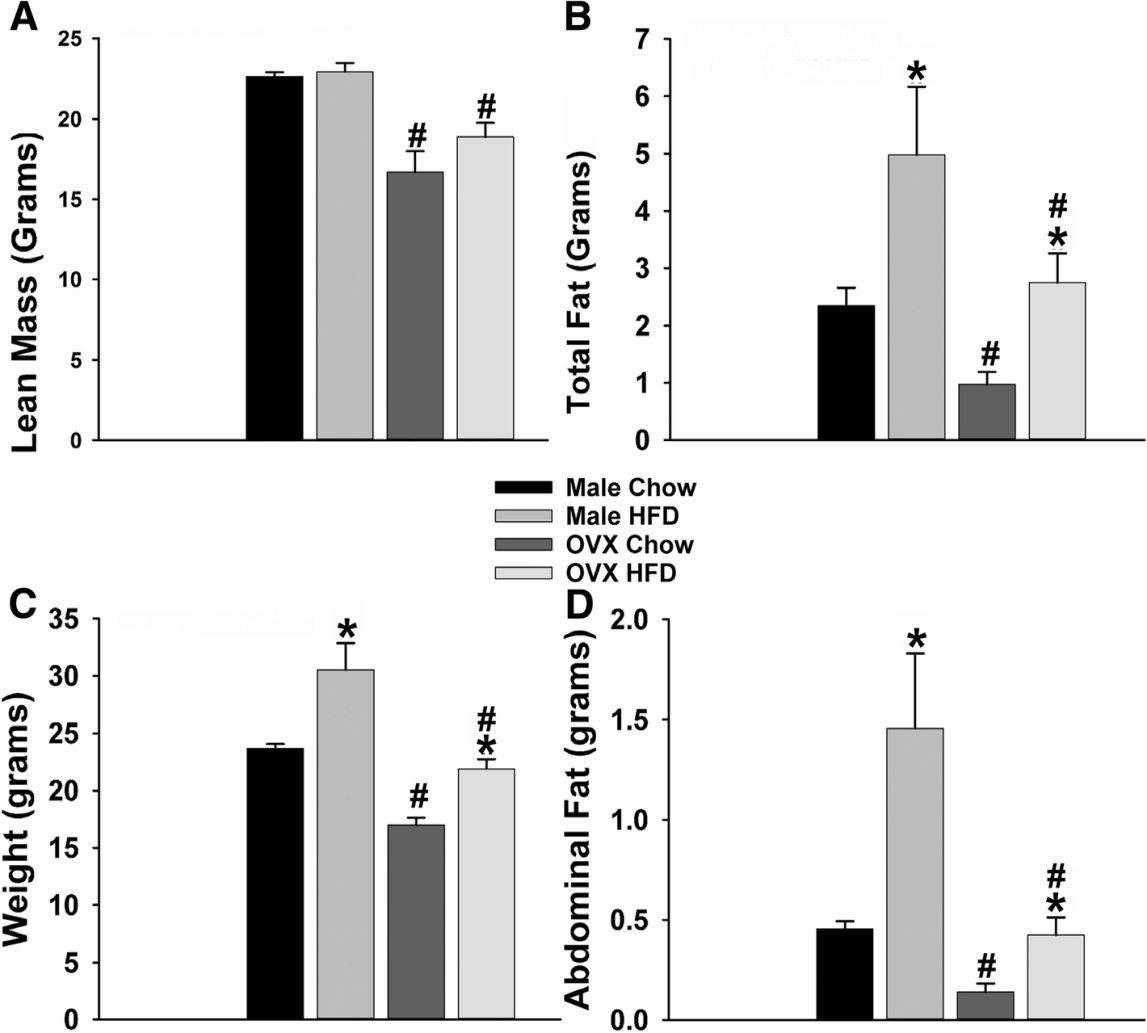
HFD-fed males and females exhibit greater body weight and adiposity than their chow-fed counterparts. Average lean mass (**a**) in intact male chow-fed (*n* = 14) and high-fat fed (*n* = 13) as well as OVX chow-fed (*n* = 13) and HFD-fed females (*n* = 14) animals along with total fat (**b**), average body weight (**c**), and average abdominal fat (**d**) in grams. Lean mass and total fat were determined via MRI. Body weight was recorded on the day of the terminal harvest, and fat dissections were performed during harvest by making an incision through the umbilical region of the abdomen. Fat was collected from the dorsolumbar, inguinal, gluteal, epididymal, and perirenal regions of the abdomen and pelvic area. Bars represent means, and vertical lines 1 SEM of the lean mass (**a**), total fat (**b**), body weight (**c**), and fat mass (**d**). *p < 0.05 relative to standard chow; multi-factorial ANOVA/LSD; #*p* < 0.05 relative to males; multi-factorial ANOVA/LSD

NR5A1-Cre and eGFP-POMC mice were randomly subdivided and given continuous access to either a standard rodent chow (Teklad Rodent Diet, Teklad Diets, Madison, WI, USA) from which 18% of the calories were derived from fat, 24% from protein, and 58% from carbohydrates or a HFD (Research Diets, New Brunswick, NJ, USA) from which 45% of calories were derived from fat, 20% from protein, and 35% from carbohydrates. All animals were kept on their respective diets for a minimum of 5 weeks prior to experimentation, after which both male and female HFD-fed mice showed a clear obese phenotype (Fig. 1a: multi-factorial ANOVA/LSD: *F*_diet_ = 3.03 (df = 1, *p* < 0.100), *F*_sex_ = 37.64 (df = 1, *p* < 0.0001), *F*_interaction_ = 0.02 (df = 1, *p* < 0.90); Fig. 1b: multi-factorial ANOVA/LSD: *F*_diet_ = 6.98 (df = 1, *p* < 0.020), *F*_sex_ = 6.19 (df = 1, *p* < 0.020), *F*_interaction_ = 2.21 (df = 1, *p* < 0.15); Fig. 1c: multi-factorial ANOVA/LSD: *F*_diet_ = 19.37 (df = 1, *p* < 0.0001), *F*_sex_ = 34.86 (df = 1, *p* < 0.0001), *F*_interaction_ = 2.05 (df = 1, *p* < 0.20); Fig. 1d: multi-factorial ANOVA/LSD: *F*_diet_ = 12.79 (df = 1, *p* < 0.0010), *F*_sex_ = 13.97 (df = 1, *p* < 0.0005), *F*_interaction_ = 4.85 (df = 1, *p* < 0.6000)) that is associated with overt insulin resistance and glucose intolerance [38].

### Surgical procedures

For all surgeries, animals were administered carprofen (Vetranal by Sigma Aldrich, 5 mg/mL; give 5 mg/kg; s.c.; both preemptively and 1 day post-surgery) to mitigate against surgical and postoperative pain, as well as sulfamethoxazole/trimethoprim suspended in their drinking water (0.48 g/L) in order to minimize the potential for postoperative infections. For some experiments, both NR5A1-Cre and eGFP-POMC female mice were OVX while they were under 2% isoflurane anesthesia. In order to focally inject adeno-associated viral vector (AAV) constructs, NR5A1-Cre mice were anesthetized with 2% isoflurane and placed in a stereotaxic frame. An incision was made to expose the skull, and a single hole was drilled on one side of the mid-sagittal suture so that an injection needle could be slowly lowered into the dorsomedial subdivision of the VMN (coordinates from bregma: AV − 0.6 mm, ML ± 0.3 mm, and DV − 5.6 mm from dura). A unilateral injection of a Cre-recombinase-dependent AAV vector containing cation channel rhodopsin-2 (ChR2; AAV1.EF1a.DIO.ChR2 (E123A).YFP.WPRE.jGH; 7.2 × 10^12^ genomic copies/mL; 300 nL total volume; University of Pennsylvania Vector Core; Addgene plasmid #35507) was given over 2 min. The injection needle remained in place for 10 min after infusion to allow for diffusion from the tip and was then slowly removed from the brain to reduce potential spread of the virus from the desired anatomical location. Animals were used for experimentation 2–3 weeks after viral injection and 1–2 weeks after OVX. Only those animals that (a) showed clear evidence of accurate AAV injection in the VMN (as indicated by the fluorescence of the YFP reporter) and (b) maintained their bright, alert, and responsive status and regained a positive growth trajectory post-surgery were included in the present study.

The stereotaxic implantation of a guide cannula into the ARC of the mice was performed similar to that described above. Briefly, once anesthetized, an animal was secured in a stereotaxic frame (Stoelting, Wood Dale, IL, USA), and a midline incision was made through the scalp. A hole was then drilled in the skull, through which a 26-gauge guide cannula (Plastics One, Roanoke, VA, USA) was lowered 1 mm above the ARC using the following coordinates: AP − 0.6 mm, ML − 0.3 mm, DV − 4.9 mm. The guide cannula was fastened in place with C&B Metabond dental cement (Parkell, Edgewood, NY, USA) applied to the surgical field. Finally, a stylet was inserted into the guide cannula to keep the lumen patent. The animals were allowed to recover for 1 week prior to the start of experimentation. Only those animals in which we could verify accurate guide cannula placement within the ARC were included in this study.

### Drugs

All drugs were purchased from Tocris Bioscience/R&D Systems (Minneapolis, MN, USA) unless otherwise stated. For electrophysiological experiments, the GABA_A_ receptor antagonist 6-imino-3-(4-methoxyphenyl)-1(6H)-pyridazinebutanoic acid hydrobromide (SR 95531) was dissolved in Ultrapure H_2_0 to a stock concentration of 10 mM, and the stock solution was diluted further with artificial cerebrospinal fluid (aCSF) to the working concentration of 10 μM. N/OFQ was prepared as a 1 mM stock solution in UltraPure H_2_0 and diluted further with aCSF to the working concentration of 1 μM. The NOP receptor antagonist (2R)-1-(phenylmethyl)-*N*-[3-(spiro[isobenzofuran-1(3H),4′-piperidin]-1-yl)propyl-2-pyrrolidinecarboxamide (BAN ORL 24 (BAN)) was prepared as a 10 mM stock solution in UltraPure H_2_O and diluted further with aCSF to the working concentration of 10 μM. The Na^+^ channel blocker octahydro-12-(hydroxymethyl)-2-imino-5,9:7,10a-dimethano-10aH-[1,3]dioxocino[6,5-d]pyrimidine-4,7,10,11,12-pentol (tetrodotoxin, TTX) was prepared as a 1 mM stock solution in UltraPure H_2_0 and diluted further with aCSF to the working concentration of 500 nM. 1, 3, 5(10)-Estratrien-3, 17β-diol (17β-estradiol (E_2_); Steraloids, RI, USA) was dissolved in punctilious ethanol to a stock concentration of 1 mM, which was further diluted to a working concentration of 100 nM. All aliquots of the stock solutions were stored at either four or − 20 °C until needed for experimentation.

For all behavioral experiments, N/OFQ was prepared as a 1.5 mM stock solution by dissolving it in filtered saline and injected directly into the ARC at a 0.3 nmol dose. Estradiol benzoate (EB; Steraloids, Newport, RI, USA) was initially prepared as a 1 mg/mL stock solution in punctilious ethanol. A known quantity of this stock solution was added to a volume of sesame oil sufficient to produce a final concentration of 100 μg/mL following evaporation of the ethanol.

### Hypothalamic slice preparation

On the day of experimentation, the animal was briefly anesthetized with 32% isoflurane and rapidly decapitated. The brain was removed from the skull, and the hypothalamic area was dissected. We then mounted the hypothalamic block on a cutting platform that, for the guinea pig, was secured in a vibratome well filled with ice-cold, oxygenated (95% O_2_, 5%CO_2_) aCSF (NaCl, 124; NaHCO_3_ 26; dextrose 10, HEPES 10; KCl 5; NaH_2_PO_4_ 2.6; MgSO_4_ 2; CaCl_2_ 1; in mM). For the mice, we used a sucrose-based cutting solution (NaHCO3 26; dextrose 10, HEPES 10; Sucrose 208; KCl 2; NaH_2_PO_4_ 1.25; MgSO_4_ 2; CaCl_2_ 1; in mM). Four to five coronal slices (300 μm) through the rostrocaudal extent of the ARC were then cut. The slices were transferred to an auxiliary chamber containing oxygenated aCSF at room temperature and maintained there until the electrophysiological recording.

### Electrophysiology

Whole-cell patch clamp electrophysiological recordings from ARC neurons using biocytin-filled electrodes were performed in hypothalamic slices prepared from gonadally intact male/female NR5A1-Cre and eGFP-POMC mice, as well as OVX female NR5A1-Cre and eGFP-POMC mice. To ascertain whether the hypothesized pleiotropic actions of N/OFQ occurred in other species, recordings in slices from male and periovulatory female guinea pigs were also conducted. During recordings, the slices were maintained in a chamber perfused with warmed (35 °C), oxygenated aCSF in which the CaCl_2_ concentration raised to 2 mM. Artificial CSF and all drugs (diluted with aCSF) were perfused via peristaltic pump at a rate of 1.5 mL/min. Patch electrodes were prepared from borosilicate glass (World Precision Instruments, Sarasota, FL, USA; 1.5 mm OD) pulled on a P-97 Flaming Brown puller (Sutter Instrument Co., Novato, CA, USA), and filled with an internal solution containing the following (in mM): potassium gluconate 128, NaCl 10, MgCl2 1, EGTA 11, HEPES 10, ATP 1, GTP 0.25, 0.5% biocytin, adjusted to a pH of 7.3 with KOH and osmolality 286–320 mOsm. Electrode resistances varied from 3 to 8 MΩ.

For guinea pig experiments, whole-cell patch clamp recordings were performed using a Multiclamp 700A preamplifier (Axon Instruments, Foster City, CA, USA) that amplified potentials and passed current through the electrode. Membrane currents were recorded in voltage clamp with access resistances ranging from 8 to 20 MΩ. The signals underwent analog-digital conversion via a Digidata 1322A interface coupled to pClamp 10.5 software (Axon instruments). For the transgenic mouse experiments, recordings were made on an Olympus BX51 W1 fixed stage microscope outfitted with infrared differential interference contrast video imaging. A Multiclamp 700B preamplifier (Molecular Devices) amplified potentials and passed current through the electrode. Membrane currents underwent analog-digital conversion with a Digidata 1550A interface (Molecular Devices) coupled to pClamp 10.5 software. The access resistance, resting membrane potential (RMP), and input resistance were monitored throughout the course of all recordings. If the access resistance deviated greater than 10% of the original value, the recording was ended. Low-pass filtering of the currents was conducted at a frequency of 2 kHz. The liquid junction potential was calculated to be − 10 mV and corrected for during data analysis using pClamp software. All recordings for the presynaptic studies were performed under a holding potential of − 75 mV, while those for the postsynaptic studies were performed at a holding potential of − 60 mV.

For the optogenetic studies described in experiments 1–3 that were designed primarily to test the hypothesis that N/OFQ decreases glutamate release at VMN SF-1/ARC POMC synapses in a sex- and diet-dependent manner, recordings were performed in slices from NR5A1-Cre mice that were injected with a ChR2-containing viral vector into the VMN 2–3 weeks prior to experimentation. Once glutamatergic SF-1-expressing fibers (visualized with YFP) impinging on ARC neurons were encountered, functional synaptic connectivity was ascertained by applying a photo-stimulus (25–100-ms pulses delivered every 2 s) from a light-emitting diode (LED) blue light source (470 nm) controlled by a variable 2A driver (ThorLabs, Newton, NJ, USA) that directly delivered the light path through the Olympus 40X water-immersion lens to generate a fast excitatory postsynaptic current (EPSC) as described previously [21]. We then determined whether the neuron under consideration was a likely POMC neuron by prescreening for intrinsic membrane currents like the A-type K^+^ current and the hyperpolarization-activated mixed cation current [37, 39, 40] via the current-voltage (I/V) relationships generated as described in the next paragraph. Baseline light-evoked excitatory postsynaptic currents (leEPSCs) were generated in the presence of the GABA_A_ receptor antagonist SR95531 (10 μM), either alone or with the NOP receptor antagonist BAN (10 μM), by photostimulating the SF-1 neurons from a holding potential of − 75 mV. After generating baseline leEPSCs over three to five 10-sweep trials, we would then co-apply N/OFQ (1 μM) amidst SR95531 with or without BAN for an additional 4–6 min, after which we would then generate leEPSCs in the presence of N/OFQ over another three to five 10-sweep trials. The N/OFQ was then allowed to clear the slice for 10 min before generating washout leEPSCs over another three to five 10-sweep trials. To analyze the leEPSCs collected prior to and in the presence of N/OFQ, alone and in conjunction with BAN, we would measure the amplitude of each leEPSC for each sweep in every trial and generate an average.

**Fig. 2:**
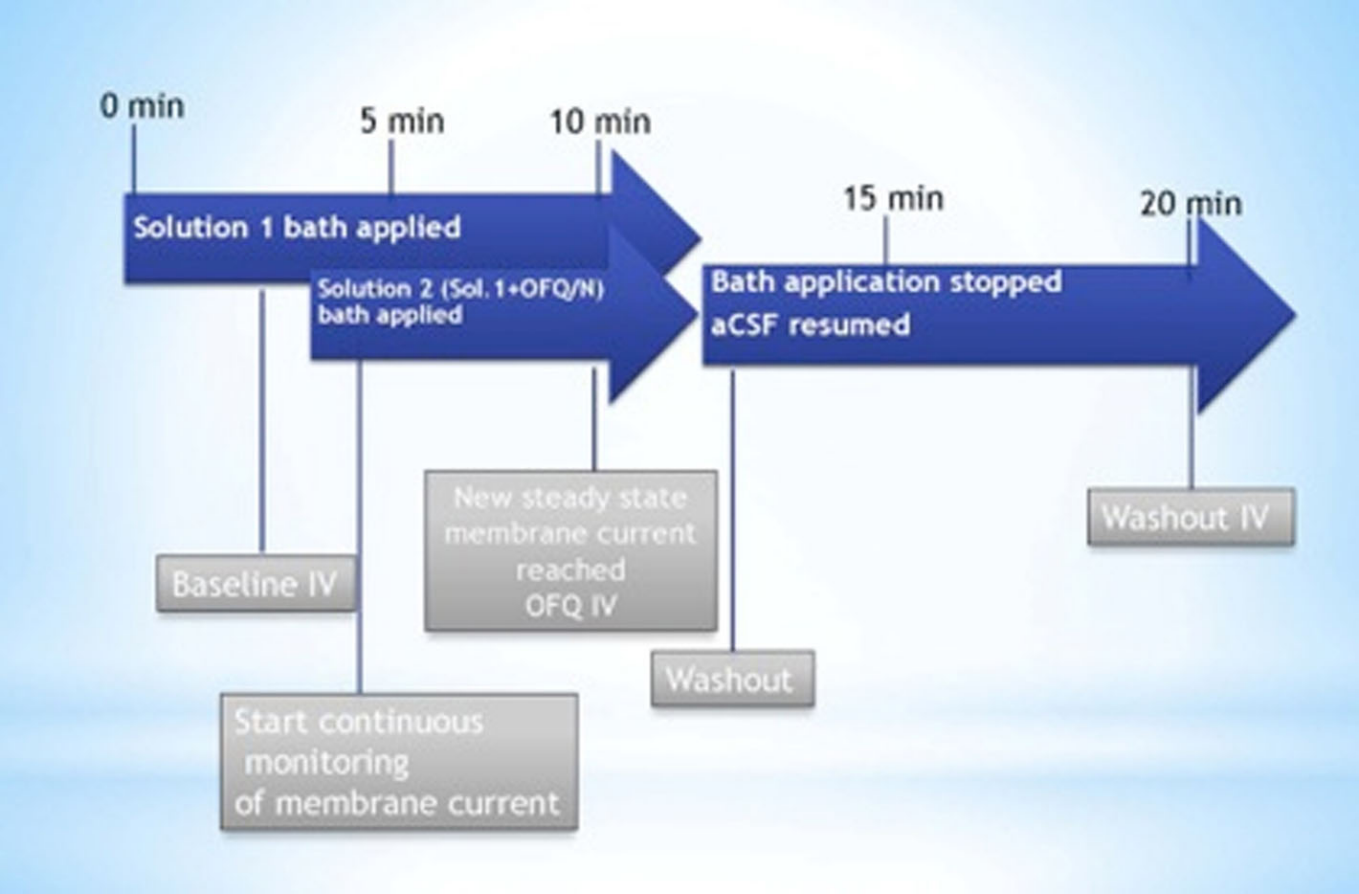
Schematic representation of drug solution perfusion protocol used during electrophysiological recordings. Solution 1 can contain either TTX, SR 95531 or nothing at all, alone or in conjunction with either BAN or E_2_

For the other experiments described in experiments 4–7 that were designed to assess the postsynaptic effect of N/OFQ, we first generated a baseline I/V relationship from a holding potential of − 60 mV by administering pulses (10 mV increments; 150 msec duration) ranging from − 50 to − 130 mV. For voltage clamp experiments, baseline I/V relationships were generated in the presence of 500 nM TTX. After the baseline I/V, N/OFQ (1 μM) is added along with TTX, and the membrane current was continuously monitored until a new steady-state value is reached, at which time a second I/V relationship is generated. During the N/OFQ washout, the membrane current (or potential) was again monitored until it returns to its original baseline level, at which time a final I/V relationship was taken to ensure reversibility of the N/OFQ-induced effect. For current clamp experiments, the membrane potential and firing rate were monitored from rest in the absence of TTX until new N/OFQ-induced steady-state levels were achieved and then monitored for an additional 10–20 min to allow for the return to baseline levels. To determine if these postsynaptic effects were NOP receptor-mediated and negatively modulated by estradiol, these same recordings were performed in slices pre-treated with 10 μM BAN or 100 nM E_2_, respectively. A schematic of the protocol for the drug administration is shown in Fig. 2.

### Immunohistochemistry

When necessary, slices were then processed after recording for immunohistochemistry using various phenotypic markers of ARC POMC neurons. Slices were initially fixed with 4% paraformaldehyde in Sorenson’s phosphate buffer (pH 7.4) for 120–180 min. They were then immersed overnight in 20% sucrose dissolved in Sorensen’s buffer and frozen in Tissue-Tek embedding medium (Miles, Inc., Elk-hart, IN, USA) the next day. Coronal sections (20 μm) were cut on a cryostat and mounted on chilled slides. These sections were then washed with 0.1 M sodium phosphate buffer (pH 7.4) and then processed with streptavidin-Alexa Flour (AF) 546 (Molecular Probes, Inc., Eugene, OR, PA, USA) at a dilution of 1:600. After localizing the biocytin-filled neuron via fluorescence microscopy, the appropriate sections were processed further with polyclonal antibodies directed against α-melanocyte-stimulating hormone (α-MSH, Immunostar, Inc., Hudson, WI, USA; 1:200 dilution), β-endorphin (Immunostar, Inc.; 1:400 dilution), cocaine and amphetamine-regulated transcript (CART; Phoenix Pharmaceuticals, Inc., Burlingame, CA, USA; 1:200 dilution), or SF-1 (Abcam, Cambridge, MA, USA; 1:300), and again evaluated using fluorescence immunohistochemistry.

### Feeding and metabolic studies

The feeding and metabolic studies were performed using a four-station Comprehensive Lab Animal Monitoring System (CLAMS; Columbus Instruments, Columbus, Ohio, USA) from which we monitored cumulative food intake, meal size, meal frequency, rate of consumption and several measures of energy expenditure (O_2_ consumption, CO_2_ production, and respiratory exchange ratio (RER) and metabolic heat production as described and validated previously [25]). These studies were conducted under conditions in which food and water were available ad libitum. The animals were allowed to acclimate in their CLAMS chamber over a 3-day period. Each day they were weighed, handled, and returned to their respective chambers. After the 3-day acclimation session, we initiated the 5-day monitoring phase during which the animals were weighed, injected each day at 4:00 pm (2–3 h in advance of the nocturnal peak in energy consumption) with either N/OFQ (0.3 nmol) or its 0.9% saline vehicle (0.2 μL) administered directly into the ARC. OVX females were also injected with either EB (20 μg/kg; s.c.) or its sesame oil vehicle (1 mL/kg; s.c.) every other day at the same time beginning on acclimation day 2. The mice were immediately placed back in their feeding chambers and monitoring took place continuously around the clock for the next 5 days.

Cumulative food intake was taken as the total amount of food consumed at 1, 2, and 4 h after either N/OFQ or vehicle administration. Meal size is the amount of food eaten in a given hour divided by the number of meals in that same hour. The parameters of energy intake, meal pattern, and energy expenditure were continuously written to computer via an A/D converter.

### Statistical analysis

Data were analyzed using Statgraphics software (Statgraphics Centurion XVI Version 16.1 17, Starpoint Technologies, INC.) and checked for normality using Bartlett’s test. Comparisons between two groups were made with either the Student’s *t* test (for parametric data) or the Mann-Whitney *U* test (for non-parametric data). Comparisons made between more than two groups were performed using either the one-way, multi-factorial, repeated-measures multi-factorial, or rank-transformed multi-factorial analysis of variance (ANOVA; the first three for parametric data, the last one for non-parametric data) followed by the least significant difference (LSD) test, or alternatively via the Kruskal-Wallis test followed by the median-notched box-and-whisker analysis (for non-parametric data). If a significant interaction was encountered among the experimental variables following multi-factorial analyses, we then performed a one-way ANOVA to elucidate significant differences among the various treatment groups. Differences were considered statistically significant if the alpha probability was 0.05 or less.

## Results

### Experiment 1: N/OFQ significantly decreases optogenetically stimulated leESPC amplitude due to the activation of the NOP receptor

**Fig. 3:**
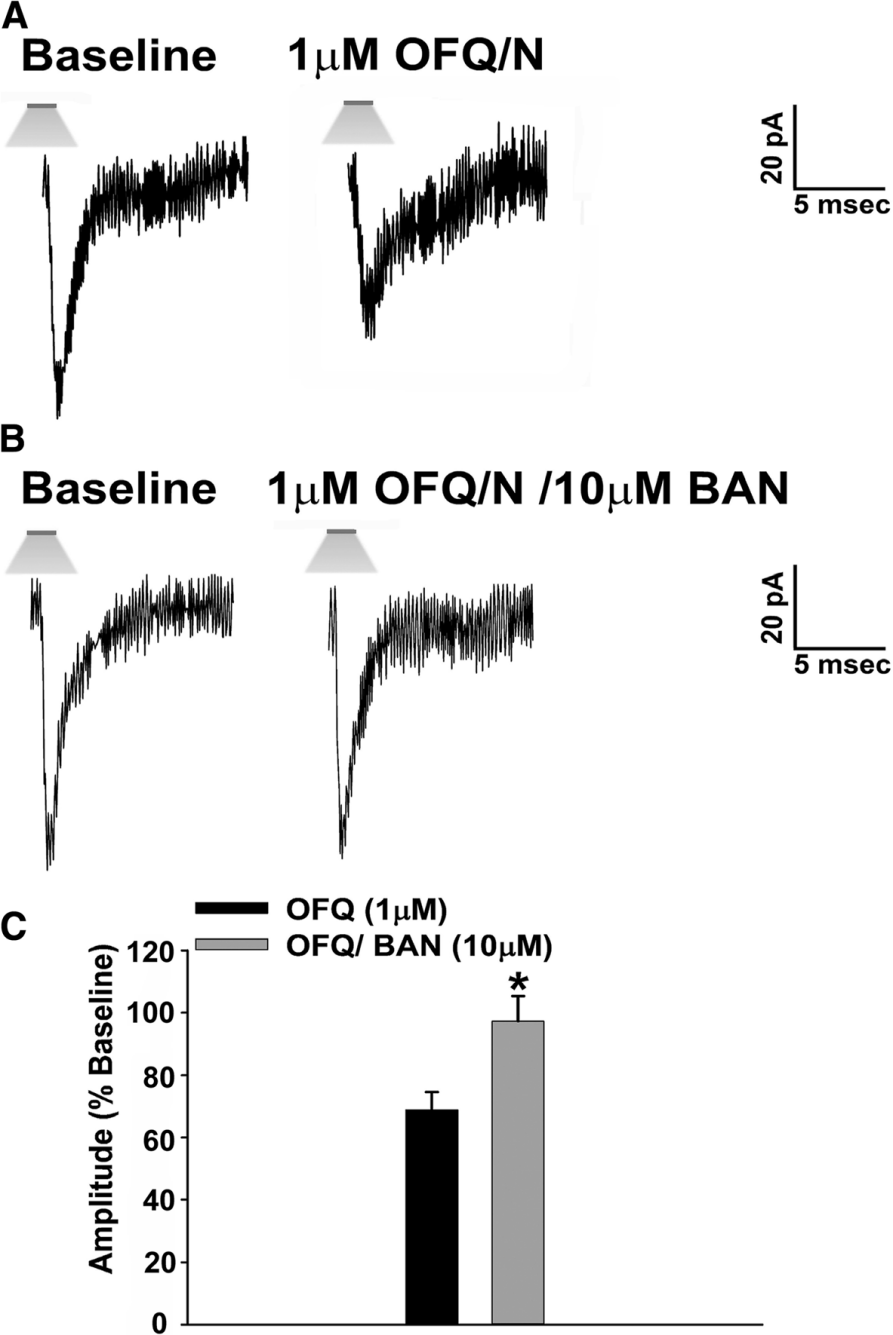
N/OFQ inhibits glutamatergic input from SF-1 neurons impinging onto POMC neurons from NR5A1-Cre mice via NOP receptor activation. The membrane current traces on the left represent baseline leEPSCs measured prior to bath application of N/OFQ, while the ones on the right were obtained in the presence of N/OFQ, alone and in the presence of the NOP receptor antagonist BAN ORL 24. **a** The N/OFQ-induced reduction in leEPSC amplitude can be seen in slices treated with N/OFQ (1 μM; *n* = 8); however, the co-application of N/OFQ and BAN abolished this effect (**b**; *n* = 5). The composite bar graph in **c** further illustrates the inhibitory effects that N/OFQ has on excitatory neurotransmission emanating from the dorsomedial VMN due to NOP activation. Bars represent means and vertical lines 1 SEM of the leEPSC amplitude in the presence of N/OFQ, alone, and in conjunction with BAN, which was normalized to that observed under baseline control conditions. *p < 0.05; Mann-Whitney *U* test

**Fig. 4:**
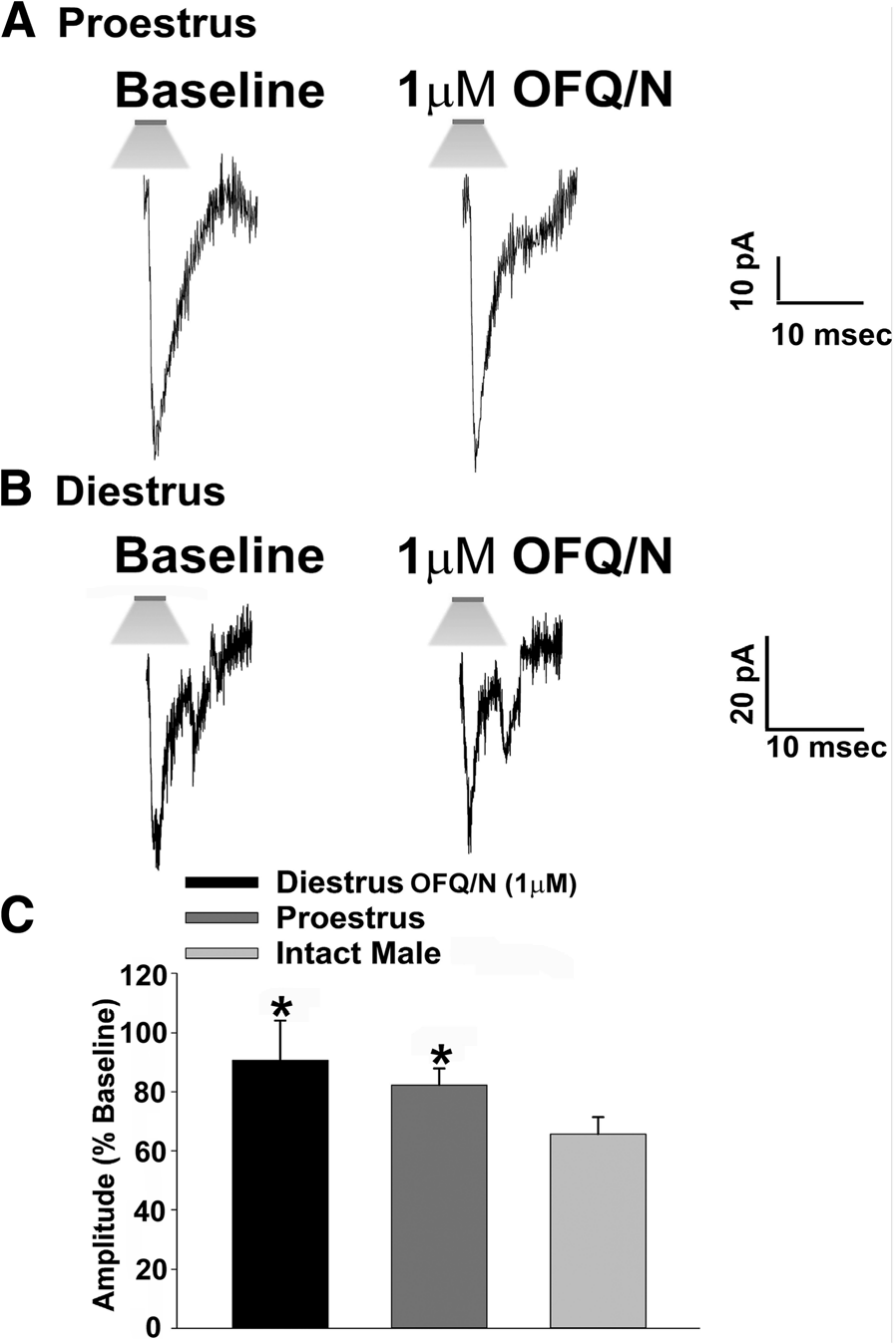
The N/OFQ-induced decrease in leEPSC amplitude at SF-1/POMC synapses in NR5A1-Cre mice is sexually differentiated. The membrane current traces on the left are representative baseline leEPSCs before the application of N/OFQ, while the ones to the right are indicative of leEPSCs in the presence of N/OFQ. The N/OFQ-induced reduction in leEPSC amplitude during different stages of the estrous cycle can be seen in slices taken from a proestrus female (**a**; *n* = 12) and a diestrus female (**b**; *n* = 5). Note that the N/OFQ-induced decrease in leEPSC amplitude seen in **a** and **b** is comparatively smaller than seen in males. The composite bar graph in (**c**) further illustrates the sex-dependent NO receptor-mediated reduction in the amplitude of excitatory input from the dorsomedial VMN onto POMC neurons. Bars represent means, and vertical lines 1 SEM. *p < 0.05; Kruskal-Wallis/median-notched box-and-whisker plot

Previous studies have shown that N/OFQ decreases glutamatergic input onto POMC neurons in the ARC [36]. It has also been shown that a possible source of this input is from SF-1 neurons located in the VMN [18–20, 22]. In order to examine if N/OFQ can decrease glutamatergic input onto POMC neurons via NOP activation, we evoked EPSCs generated by light stimulation of SF-1 neurons located in the dorsomedial VMN. We recorded a total of 44 ARC POMC neurons from NR5A1-Cre mice. These animals express a cre-recombinase controlled by the NR5A1 promoter, which is the gene that encodes for the SF-1 transcription factor. They were injected directly into the VMN with an AAV construct containing ChR2 tagged with a YFP reporter (as previously mentioned) in order to activate the SF-1 neurons with photostimulation. We then performed visualized, whole-cell patch clamp recordings in ARC neurons 2 to 3 weeks later, which were later identified as POMC neurons via immunohistochemistry (Additional file 1: Figure S1A–F). Optogenetic stimulation elicited robust leEPSCs (Fig. 3) that, in some cases, resulted in a second EPSC appearing before the first response had completely decayed (Fig. 4). N/OFQ (1 μM) significantly decreased leEPSC amplitude in intact males upon light stimulation (Fig. 3a, c; Mann-Whitney *U* test, *W* = 22.0, *p* < 0.050), and this effect is abolished in the presence of the NOP antagonist BAN (10 μM; Fig. 3b, c; Mann-Whitney *U* test, *W* = 22.0, *p* < 0.050). These results provide a clear indication that N/OFQ inhibits glutamatergic input emanating from SF-1 neurons in the dorsomedial VMN due to the activation of the NOP receptor.

### Experiment 2: The N/OFQ-induced decrease in glutamatergic input onto POMC neurons due to NOP activation is sexually differentiated

Now that we know that the N/OFQ-induced decrease in glutamatergic input onto ARC POMC neurons emanating from SF-1 neurons in the dorsomedial VMN is due to the activation of the NOP receptor, we next wanted to examine if this action is sexually differentiated. Figure 4 shows the representative basal leEPSCs for NR5A1-Cre females during diestrus and proestrus. The degree of the N/OFQ-induced reduction in leEPSC amplitude seen in proestrus (Fig. 4a) and diestrus (Fig. 4b) is significantly smaller than that seen in the previous figure with the intact male recording (Figs. 3a, 4c; Kruskal-Wallis/median-notched box- and- whisker plot, test statistic = 11.1162, *p* < 0.005). Data from estrus and metestrus females are not included here due to the lack of functional synapses capable of generating a leEPSC even under baseline conditions. This indicates that the N/OFQ-induced decrease in excitatory neurotransmission at VMN SF-1/ARC POMC synapses is sexually differentiated, with males being more sensitive than females during certain stages of the estrous cycle.

### Experiment 3: Long-term exposure to HFD further accentuated the N/OFQ-induced decrease in leEPSC amplitude

**Fig. 5:**
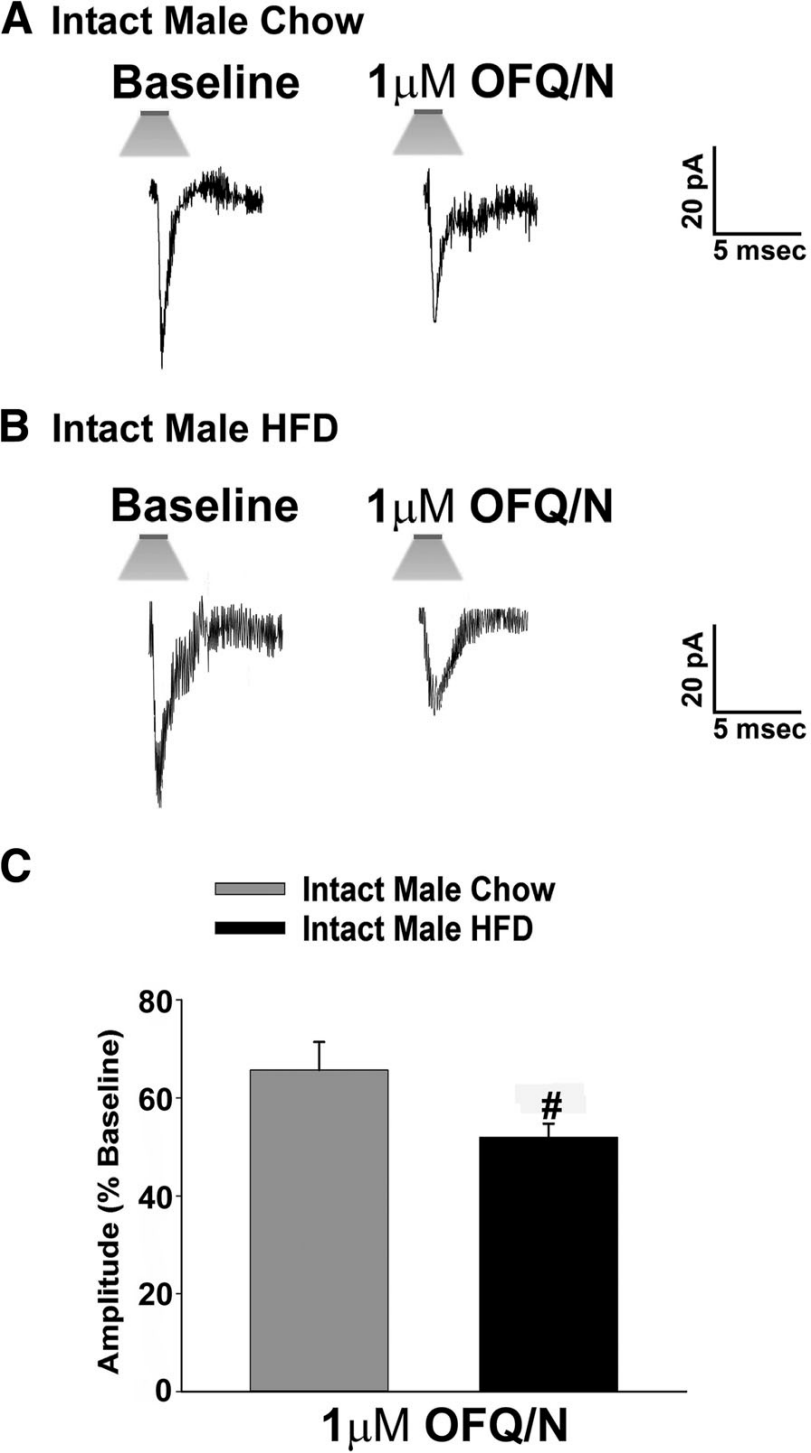
Long-term HFD exposure accentuates the N/OFQ-induced reduction in glutamatergic input at VMN SF-1/ARC POMC synapses in NR5A1-Cre mice. The membrane current traces on the left represent baseline leEPSCs before N/OFQ application, while the ones on the right were obtained in the presence of N/OFQ. The dietary modification of the N/OFQ-induced decrease in leEPSC amplitude can be seen in slices taken from a normal chow intact male (**a**; *n* = 8) and a HFD-fed intact male (**b**; *n* = 14), where the degree of the N/OFQ-induced decrease in leEPSC amplitude is significantly greater. The composite bar graph in **c** further illustrates the alterations of the N/OFQ-induced decrease in excitatory neurotransmission emanating from the dorsomedial VMN onto ARC POMC neurons caused by the HFD. Bars represent means, and vertical lines 1 SEM. *p < 0.05; Mann-Whitney *U* test

We have shown previously that long-term exposure to HFD leads to a potentiation of the CB1 receptor-mediated inhibition of glutamatergic transmission at VMN SF-1/ARC POMC synapses in male but not female mice [21]. We then wanted to see if long-term exposure to HFD could also potentiate the NOP receptor-mediated reduction in glutamatergic input at these synapses. Due to the fact that HFD-fed females do not exhibit functional glutamatergic synapses between these two cells [21], this experiment was performed only in males. Indeed, N/OFQ-induced decrease in leEPSC amplitude in POMC neurons from these animals was further accentuated by the long-term HFD exposure in comparison to that seen in the chow-fed males (Fig. 5a–c; Mann-Whitney *U* test, *W* = 16.0, *p* < 0.050). These data indicate that long-term HFD exposure augments the N/OFQ-induced inhibition of excitatory neurotransmission at anorexigenic VMN SF-1/ARC POMC synapses.

### Experiment 4: N/OFQ postsynaptically inhibits POMC and SF-1 neurons by inducing an outward current due to the activation of the NOP receptor

**Fig. 6:**
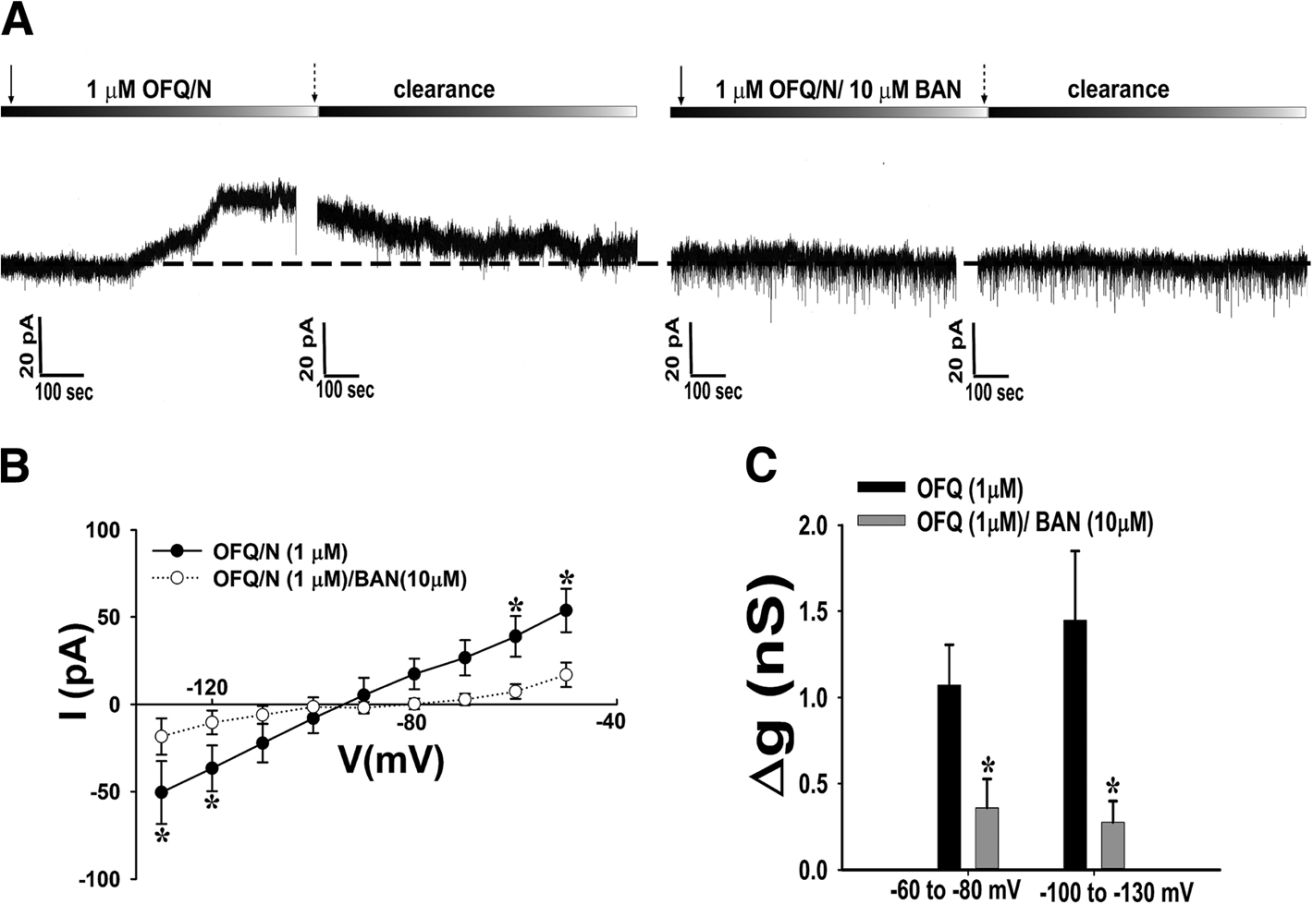
N/OFQ produces a reversible NOP receptor-mediated outward current due to activation of GIRK channels in eGFP-POMC mice. **a** Membrane current traces show the reversible outward current caused by 1 μM N/OFQ (*n* = 14), and the abolition of the outward current in the presence of BAN (*n* = 16). Composite I/V plot (**b**) and bar graph (**c**) illustrating the marked reduction in the N/OFQ- induced increase in slope conductance caused by BAN. Symbols represent means of the membrane current (*I*) seen at different command voltages (*V*), whereas bars represent the change in slope conductance (Δg) measured via linear regression between − 60 to − 80 mV and − 100 to − 130 mV, in the presence of N/OFQ alone or with BAN. Vertical lines indicate 1 SEM. *p < 0.05, multi-factorial ANOVA/LSD

**Fig. 7:**
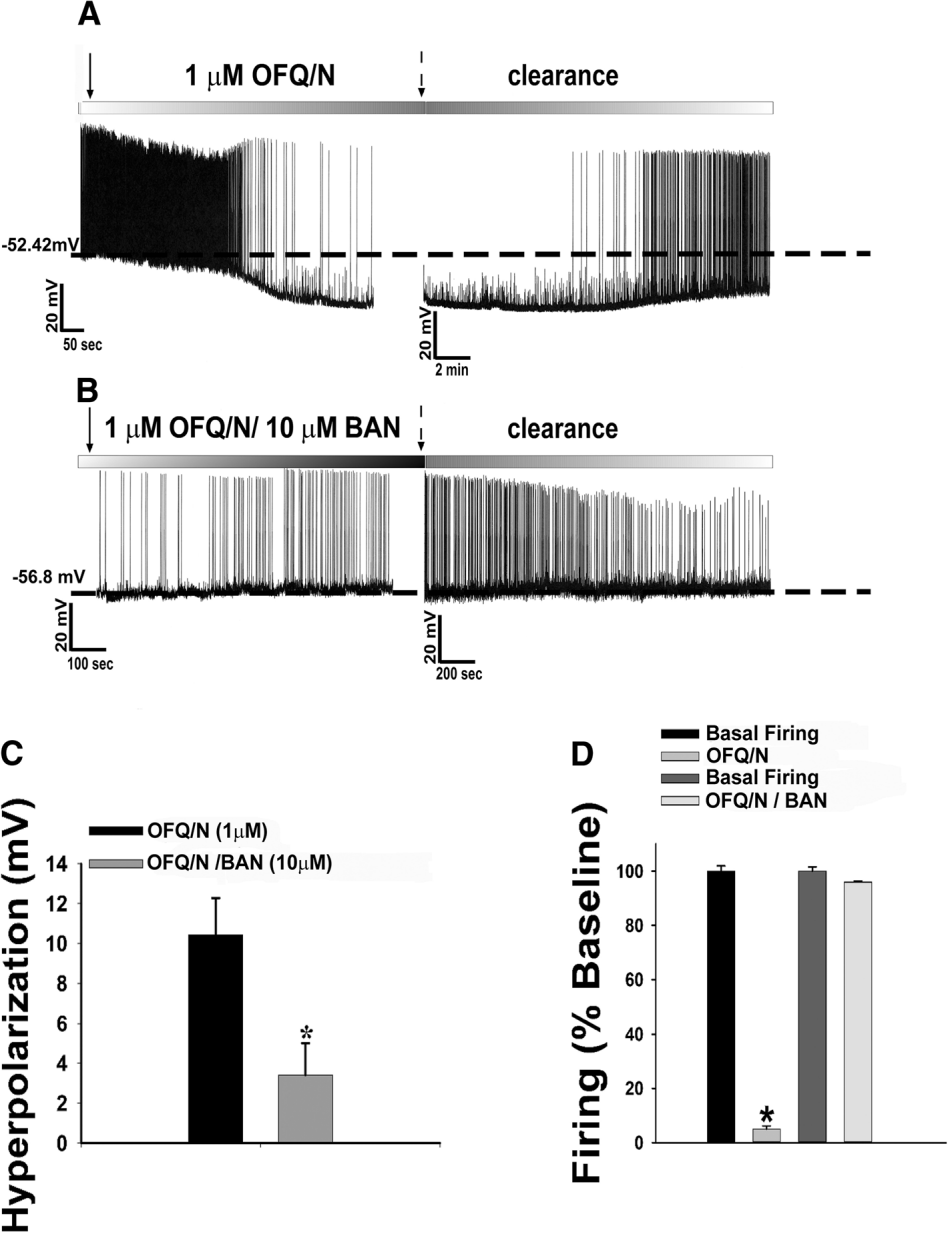
N/OFQ reversibly hyperpolarizes and decreases firing in POMC neurons from eGFP-POMC mice via an NOP receptor-mediated mechanism. Membrane current traces show the reversible hyperpolarization and decrease in firing cause by N/OFQ (**a**; *n* = 13) and the abolition of these effects in the presence of BAN (**b**; *n* = 11). The composite bar graphs in (**c**, **d**) show the abrogation of N/OFQ-induced hyperpolarization and decrease in firing due to BAN application. Bars are representative of the means, while lines represent 1 SEM of the membrane hyperpolarization (**c**) and the normalized change in firing rate (**d**) caused by N/OFQ alone and in combination with BAN. *p < 0.05, Student’s *t* test (**c**) or Kruskal-Wallis/ median-notched box-and-whisker plot (**d**)

To this point, we have shown that N/OFQ activates presynaptic NOP receptors to modulate excitatory neurotransmission at VMN SF-1/ARC POMC synapses in a sex- and diet-dependent manner. We now wanted to examine the postsynaptic effects in order to better ascertain the pleiotropic actions of the peptide. We have shown previously that N/OFQ induces a robust outward current that reverses polarity around the Nernst equilibrium potential for K^+^ and is associated with an increase in conductance in ARC POMC neurons [12, 41]. Here, we recorded from 141 ARC POMC neurons in eGFP-POMC mice in order to determine if this effect is due to the activation of the NOP receptor. In keeping with previous reports demonstrating that the eGFP is expressed in the overwhelming preponderance of POMC neurons [39, 42], all 20 recorded neurons subsequently tested were immunohistochemically identified as POMC neurons (Additional file 2: Figure S2A–G). N/OFQ elicited a robust outward current in recordings taken from intact male eGFP-POMC neurons, an effect that was reversed by co-application of the NOP receptor antagonist BAN (Fig. 6a). Additionally, the composite I/V plot shows the prominent change in conductance and reversal of polarity around the equilibrium potential for K^+^ (Fig. 6b: multi-factorial ANOVA/LSD: *F*_voltage_ = 0.36 (df = 1, *p* < 0.60), *F*_OFQ_ = 14.83 (df = 1, *p* < 0.0005), *F*_interaction_ = 0.87 (df = 1, *p* < 0.40); Fig. 6c: multi-factorial ANOVA/LSD: *F*_voltage_ = 17.75 (df = 8, *p* < 0.001), *F*_OFQ_ = 0.87, (df = 1, *p* < 0.40), *F*_interaction_ = 4.05 (df = 8, *p* < 0.0005); one-way ANOVA/LSD: *F*_between groups_ = 7.55 (df = 17, *p* < 0.001)), which further illustrates the robust NOP receptor-mediated K^+^ current triggered by the activation of GIRK channels. The robust NOP receptor-mediated outward current and increase in conductance seen in males was also associated with an equally prominent, reversible hyperpolarization and decrease in firing (Fig. 7c Student’s *t* test, *t* = 2.299, *p* < 0.040; Fig. 7d Kruskal-Wallis/median-notched box-and whisker plot, test statistic = 6.75, *p* < 0.01). This effect was again completely abolished upon co-application of the NOP receptor antagonist BAN (10 μM).

### Experiment 5: The N/OFQ-induced outward current and increase in conductance is sexually differentiated

**Fig. 8:**
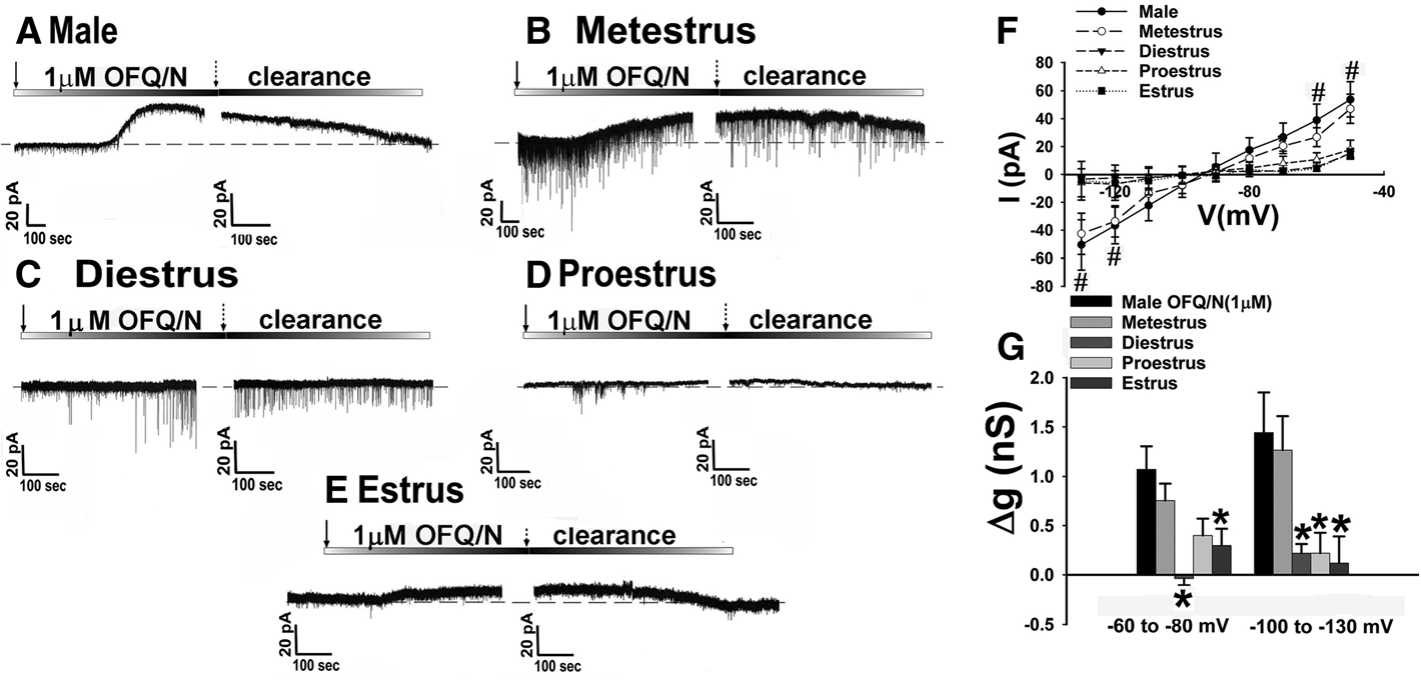
The N/OFQ-induced activation of GIRK channels in eGFP-POMC mice is sexually differentiated. Membrane current traces show the N/OFQ-induced outward current in males (**a**; *n* = 14), and the attenuation across different stages of the estrous cycle seen in diestrus (**c**; *n* = 5), proestrus (**d**; *n* = 8), and estrus (**e**; *n* = 10), but not metestrus (**b**; *n* = 10). The composite I/V plot (**f**) and bar graph (**g**) further illustrate the marked reduction in slope conductance during these particular stages of the estrous cycle. Bars and symbols represent means while the lines are indicative of 1 SEM. *p < 0.05, multi-factorial ANOVA/LSD

Since we saw previously that the presynaptic effects of NOP receptor activation are sexually differentiated, we then wanted to examine how sex modifies the postsynaptic effects of N/OFQ. As hypothesized, N/OFQ produced a robust outward current in male and metestrus female eGFP-POMC mice (Fig. 8a, b). However, in proestrus, estrus, and diestrus females (Fig. 8c–e), the N/OFQ-induced outward current was markedly attenuated. The fluctuations in the magnitude of the outward current across different stages of the estrous cycle corresponded with similar variations in the N/OFQ-induced increase in conductance (Fig. 8f: multi-factorial ANOVA/LSD: *F*_voltage_ = 11.09 (df = 8, *p* < 0.0001), *F*_sex_ = 0.08 (df = 4, *p* < 1.0), *F*_interaction_ = 2.04 (df = 32, *p* < 0.0020); one-way ANOVA/LSD: *F*_between groups_ = 5.02 (df = 44, *p* < 0.0001); Fig. 8g: multi-factorial ANOVA/LSD: *F*_voltage_ = 0.58 (df = 1, *p* < 0.50), *F*_sex_ = 6.31 (df = 4, *p* < 0.0005), *F*_interaction_ = 0.59 (df = 4, *p* < 0.70)). These results indicate that the postsynaptic effects of N/OFQ are sexually differentiated and dependent on the particular stage of the estrous cycle.

We then ventured to see if the observed sex differences in the postsynaptic effects of N/OFQ extended to other species. Here, we again performed electrophysiological recordings from ARC POMC neurons using the same whole-cell patch clamp techniques in intact male and periovulatory female guinea pigs. As expected, N/OFQ caused a pronounced hyperpolarization and a complete cessation of firing in identified POMC neurons (Additional file 3: Figure S3B: Student’s *t* test, *t* = 3.082, *p* < 0.030). More importantly, the disparity in the N/OFQ-induced outward current between male and periovulatory female guinea pigs was very similar to that of the male and female mice, in that there was a robust response and increase in conductance for the male (Additional file 4: Figure S4A), while in the female this effect was attenuated (Additional file 4: Figure S4B). The inequities in the N/OFQ-induced increase in slope conductance seen in Additional file 4: Figure S4C further illustrate the conserved nature of this sex difference in postsynaptic activation of GIRK channels in POMC neurons (multi-factorial ANOVA/LSD: *F*_voltage_ = 0.39 (df = 1, *p* < 0.60),;*F*_sex_ = 5.27 (df = 1, *p* < 0.040), *F*_interaction_ = 0.35 (df = 1, *p* < 0.60)).

### Experiment 6: Long term exposure to HFD accentuates the N/OFQ-induced outward current and increase in conductance in a sex-dependent manner

**Fig. 9:**
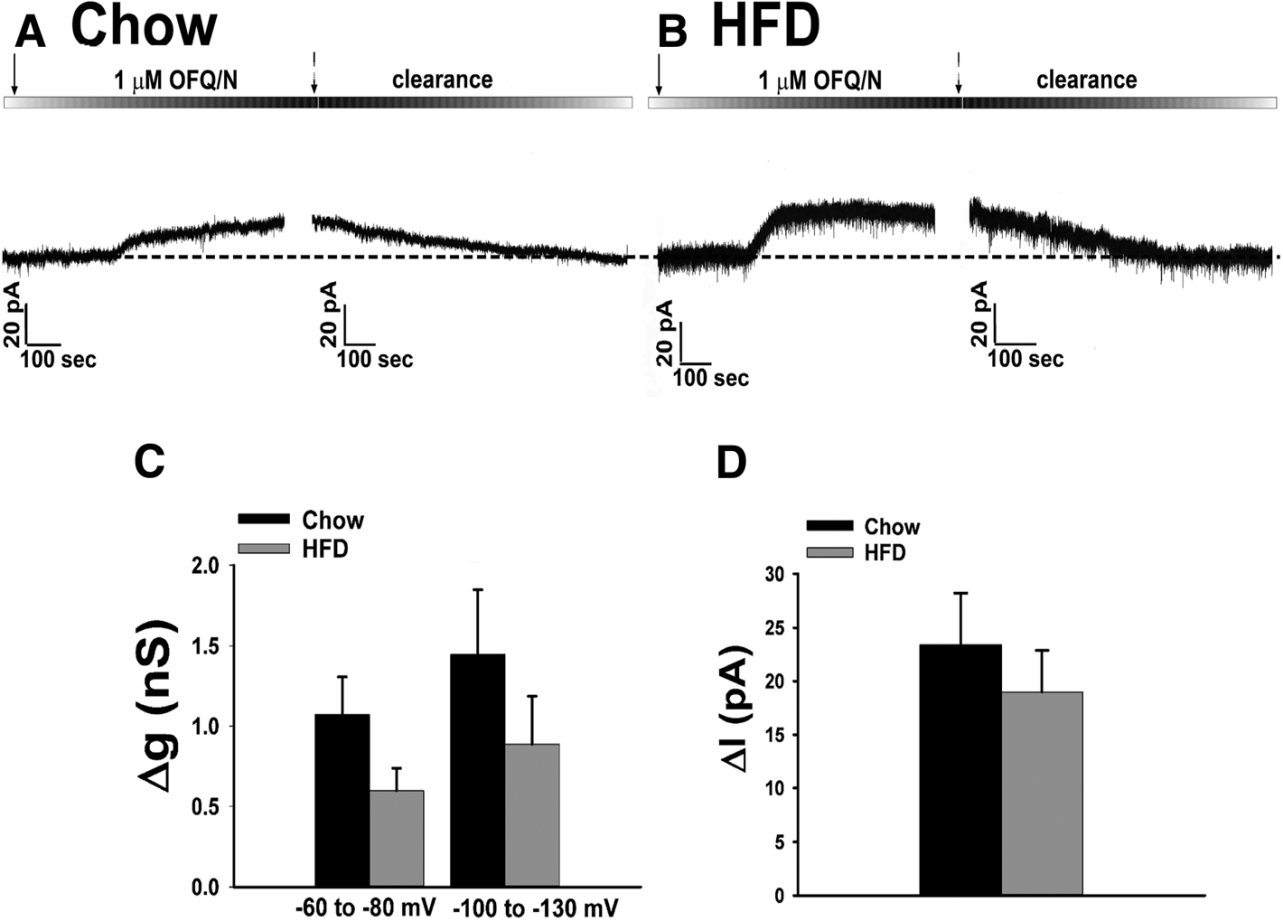
Long-term HFD exposure does not accentuate the NOP receptor-mediated activation of GIRK channels in male eGFP-POMC mice. Membrane current traces showing the N/OFQ- induced outward current seen in chow-fed (**a**; *n* = 14) and HFD-fed (**b**; *n* = 18) animals. The composite bar graph in (**c**, **d**) further illustrates the N/OFQ-induced increase in Δg seen in chow-fed and HFD-fed animals. Bars represent the means for each group while the lines are indicative of 1 SEM

**Fig. 10:**
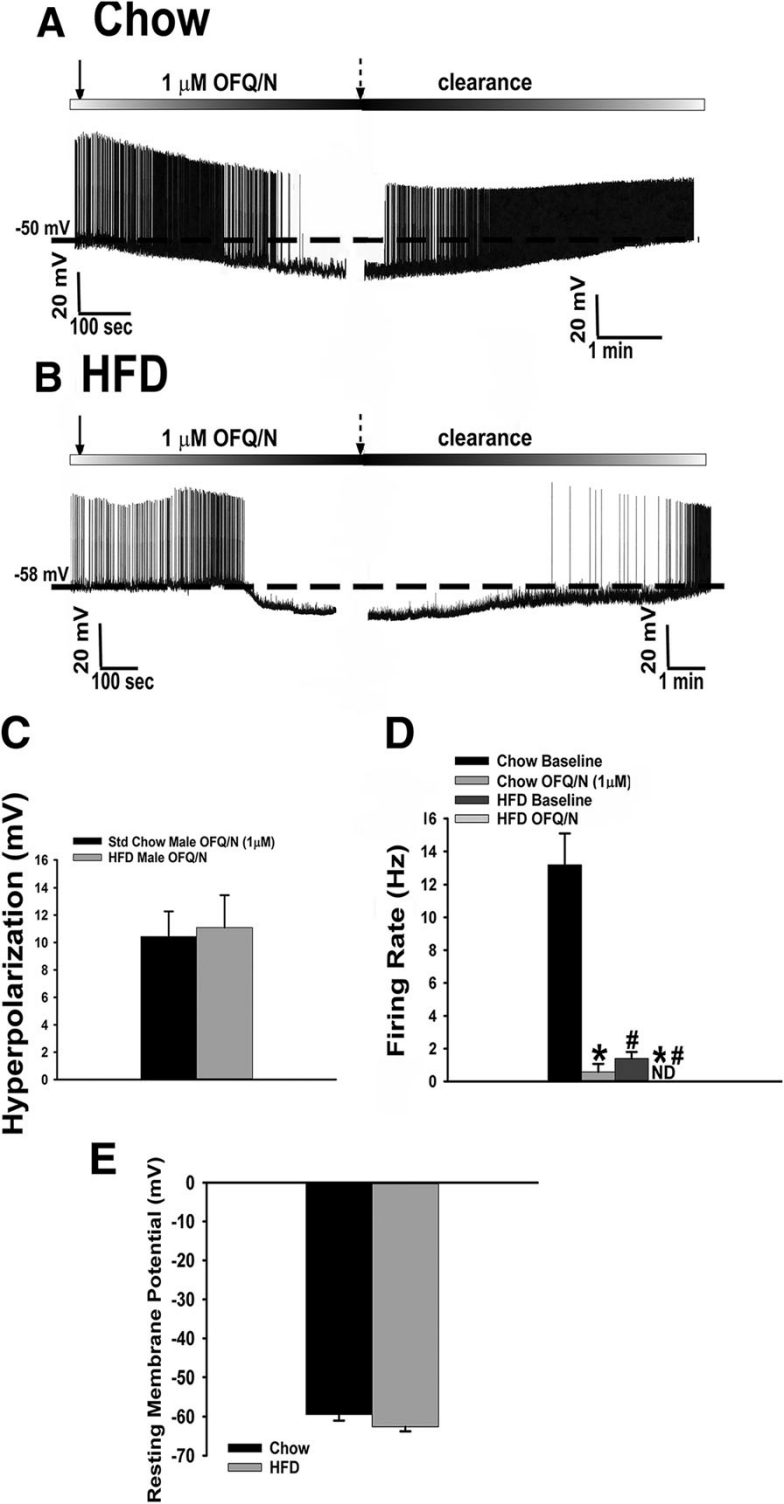
N/OFQ reversibly hyperpolarizes and decreases firing in POMC neurons in HFD-fed eGFP-POMC males. Membrane current traces showing the reversible hyperpolarization and decrease in firing caused by N/OFQ in chow-fed males (**a**; *n* = 13) and the similarity of these effects in HFD-fed males (**b**; *n* = 8). The composite bar graphs in (**c**, **d**) show the N/OFQ-induced hyperpolarization and decrease in firing in both the chow- and HFD-fed males, while the composite bar graph in **e** shows the resting membrane potential for both. Bars are representative of the means, while lines represent 1 SEM. *P < 0.05 relative to baseline, multi-factorial ANOVA/LSD. #*p* < 0.05 relative to chow, multi-factorial ANOVA/LSD

Since we saw that long-term exposure to HFD further accentuates the presynaptic effects of N/OFQ, we wanted to see if the same was true postsynaptically. Again, N/OFQ produced a robust outward current in recordings from chow-fed eGFP-POMC male mice (Fig. 9a), while paradoxically in HFD-fed males the N/OFQ-induced outward current and increase in conductance were no different (Fig. 9b, c: multi-factorial ANOVA/LSD: *F*_voltage_ = 1.39 (df = 1, *p* < 0.30), *F*_diet_ = 3.37 (df = 1, *p* < 0.080), *F*_interaction_ = 0.02 (df = 1, *p* < 0.900); Fig. 9d: Student’s *t* test: *t* = 0.735879, *p* < 0.500). The same holds true for the N/OFQ-induced hyperpolarization and cessation of firing in POMC neurons seen in these HFD-fed males (Fig. 10a–d). Interestingly, the males exposed to the HFD also had a significant decrease in their basal firing (Fig. 10d: multi-factorial ANOVA/LSD: *F*_diet_ = 31.22 (df = 1, *p* < 0.0001), *F*_OFQ_ = 40.06 (df = 1, *p* < 0.0001), *F*_interaction_ = 25.64 (df = 1, *p* < 0.0005); one-way ANOVA/LSD: *F*_between groups_ = 34.45 (df = 3, *p* < 0.0001)), as well as a slightly more hyperpolarized resting membrane potential (Fig. 10e: Student’s *t* test: *t* = 1.61033, *p* < 0.200), which could account for that previously mentioned decrease in basal firing.

**Fig. 11:**
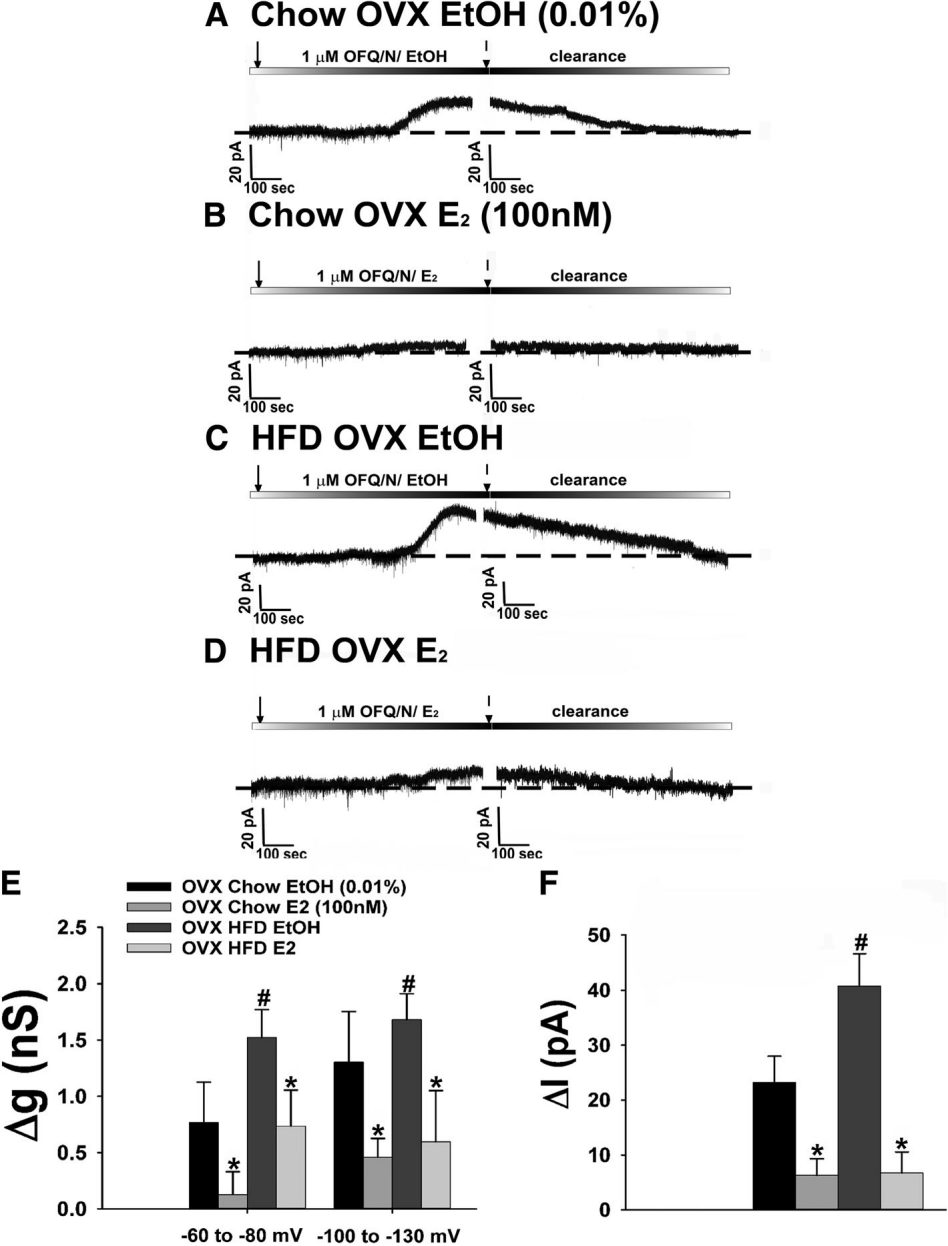
HFD accentuates whereas E_2_ attenuates N/OFQ-induced GIRK channel activation in POMC neurons from OVX eGFP-POMC females. Membrane current traces show the N/OFQ-induced outward current in chow-fed ethanol (EtOH) vehicle-treated slices (**a**; *n* = 8), which was further accentuated in HFD-fed ethanol vehicle-treated slices (**c**; *n* = 9), and the attenuation of this effect for both diets in E_2_-treated slices (**b**; *n* = 7 and **d**; *n* = 8). These effects are paralleled by similar changes in the N/OFQ-induced increase in composite slope conductance and membrane current (at − 60 mV) seen in (**e** and **f**), respectively. *p < 0.05, relative to EtOH-treated vehicle, multi-factorial ANOVA/LSD. #*p* < 0.05, relative to chow-fed controls, multi-factorial ANOVA/LSD

We also examined the postsynaptic effects of N/OFQ in OVX, chow- and HFD-fed females. Coupled with the comparatively modest N/OFQ-induced activation of GIRK channels that we saw during E_2_-dominated portions of the estrous cycle, we wanted to ascertain that it negatively modulates the effects of postsynaptic NOP receptors in this context as well. In recordings from punctilious ethanol (0.01%) vehicle-treated slices obtained from OVX, chow-fed female eGFP-POMC mice, we observed a robust outward current associated with an increase in conductance (Fig. 11a, e, f). These effects are appreciably diminished in recordings from E_2_-treated slices (100 nM; Fig. 11b, e, f). On the other hand, N/OFQ caused a comparatively more robust postsynaptic response during recordings in slices from HFD-fed females (Fig. 11c, e, f) that again was markedly attenuated in the presence of E_2_ (Fig. 11d, f, e: multi-factorial ANOVA/LSD: *F*_E2_ = 13.31 (df = 1, *p* < 0.0010), *F*_diet_ = 4.18 (df = 1, *p* < 0.050), *F*_voltage_ = 0.94 (df = 1, *p* < 0.400), *F*_E2/diet_ = 0.18 (df = 1, *p* < 0.70), *F*_E2/voltage_ = 0.30 (df = 1, *p* < 0.60), *F*_diet/voltage_ = 0.85 (df = 1, *p* < 0.40), *F*_E2/diet/voltage_ = 0.01 (df = 1, *p* < 0.95); Fig. 11f: multi-factorial ANOVA/LSD: *F*_E2_ = 29.82 (df = 1, *p* < 0.0001), *F*_diet_ = 2.95 (df = 1, *p* < 0.100), *F*_interaction_ = 5.06 (df = 1, *p* < 0.04); one-way ANOVA/LSD: *F*_between groups_ = 18.81 (df = 3, *p* < 0.0001)).

### Experiment 7: N/OFQ also produces an outward current and increases conductance in SF-1 neurons located in the dorsomedial VMN

**Fig. 12:**
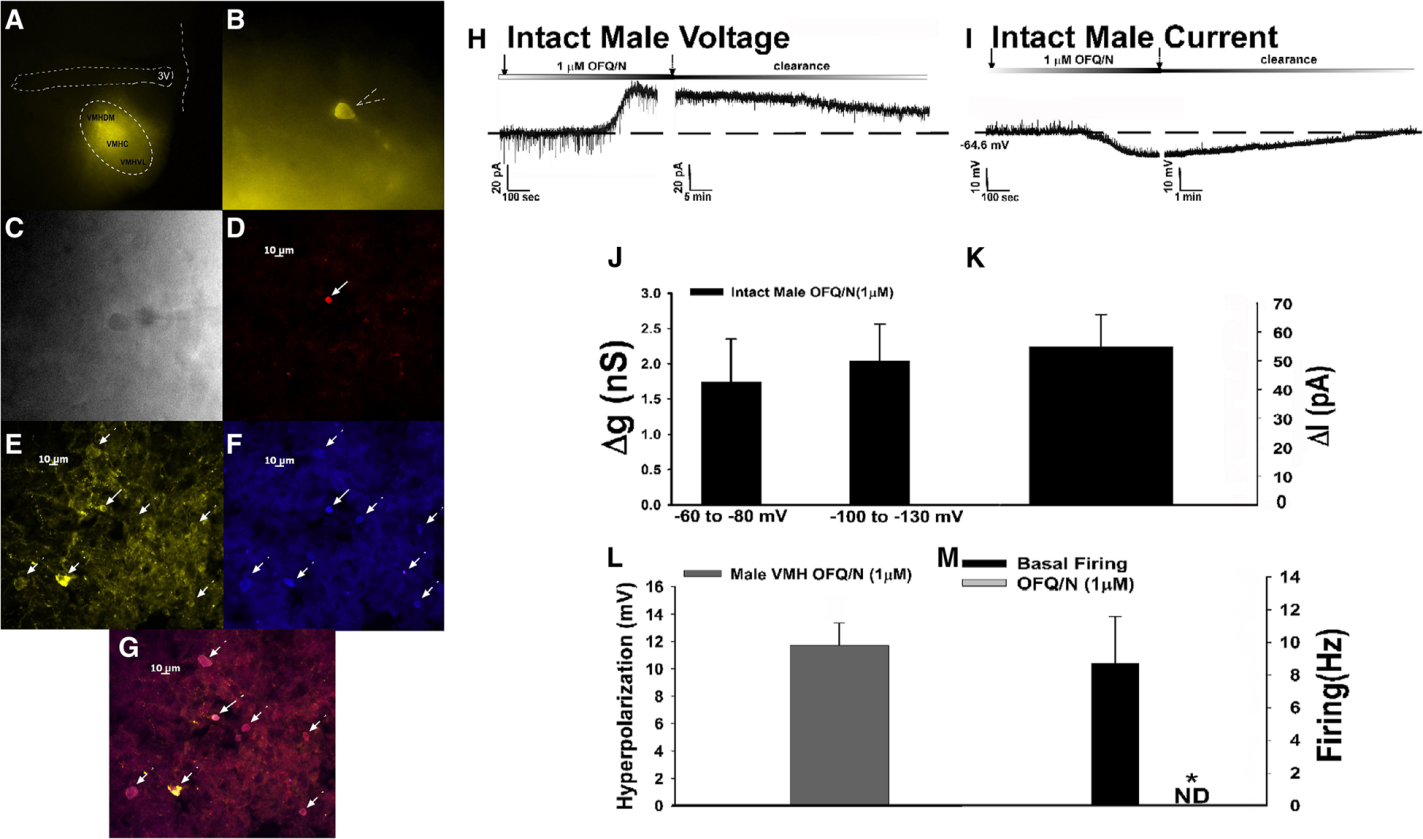
The N/OFQ-induced activation of GIRK channels occurs in SF-1 neurons located in the VMN of NR5A1-Cre mice. **a** YFP labeling of SF-1 neurons at × 4 magnification. **b** YFP labeling of the recorded SF-1 neuron at × 40 magnification. **c** DIC image of the previous neuron taken during recording. **d** Biocytin labeling of the cell in (**c**) visualized with streptavidin/AF546 as indicate by solid line. **e** YFP labeling of the same cell seen in (**b**–**d**). Surrounding YFP-filled neurons are indicated by dashed lines. **f** An antibody directed against SF-1 immunolabels the cell in **c** as visualized with AF350. **g** A composite overlay of the biocytin/YFP/SF-1 labeling seen in the cell. Panels **d–g** were photographed at × 20. The calibration bar equals 10 μm. Membrane current traces showing the N/OFQ-induced outward current (**h**; *n* = 5) and hyperpolarization (**i**; *n* = 8) in SF-1 neurons. The robust outward current is accompanied by an increase in slope conductance (**j** and **k**), and the hyperpolarization is associated with a decrease in firing (**l** and **m**). *p < 0.05, Student’s *t* test

**Fig. 13:**
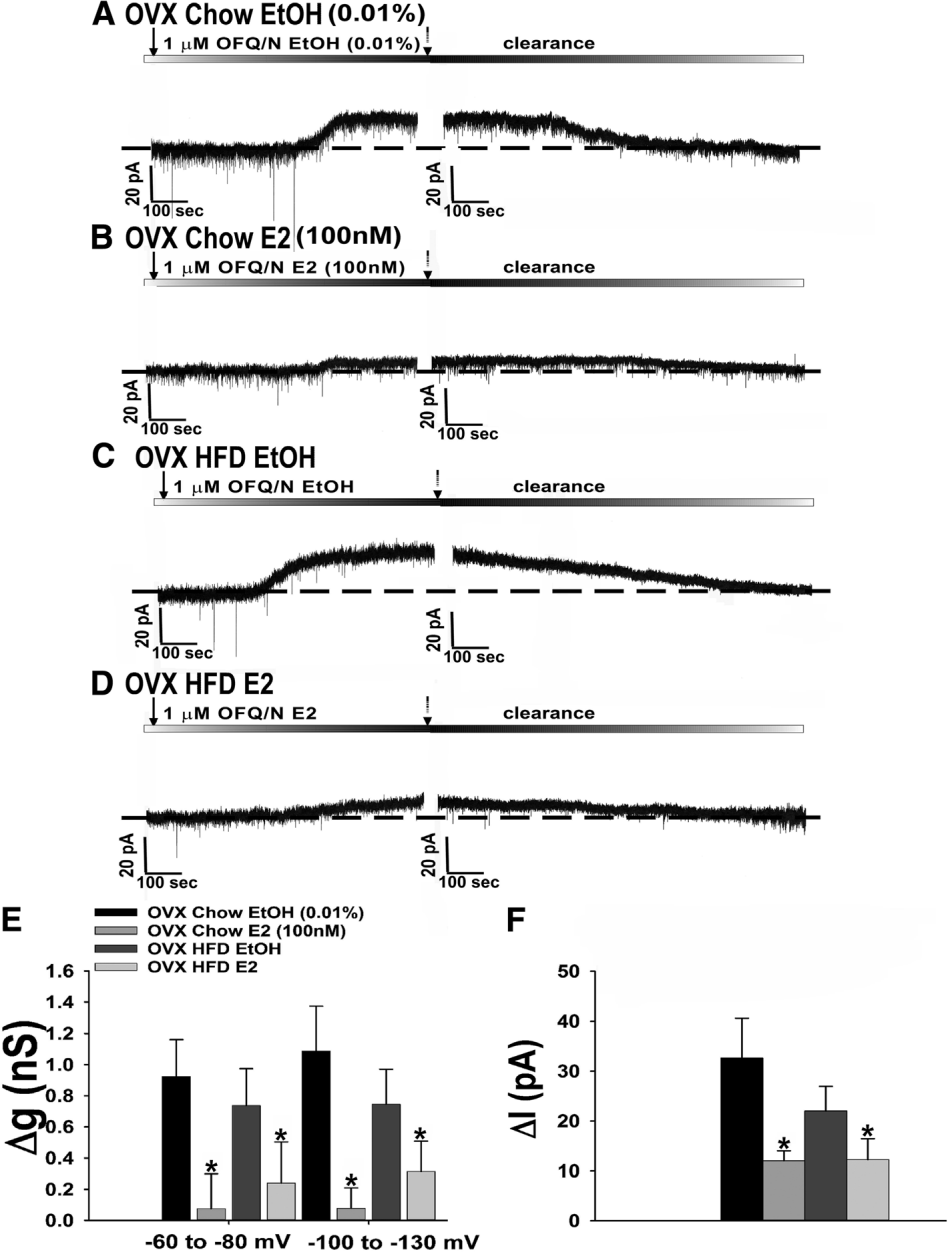
E_2_ dampens N/OFQ-induced GIRK channel activation in SF-1 neurons from chow- and HFD-fed OVX NR5A1-Cre females. Membrane current traces show the N/OFQ-induced outward current in chow-fed as well as HFD-fed ethanol vehicle-treated slices (**a**; *n* = 6 and **c**; *n* = 7) and the attenuation of this effect for both diets in E_2_-treated slices (**b**; *n* = 5 and **d**; *n* = 8). These effects are paralleled by similar diminutions in the N/OFQ-induced increase in slope conductance and membrane current (at − 60 mV) seen in (**e** and **f**)**,** respectively. *p < 0.05, relative to EtOH-treated vehicle, multi-factorial ANOVA/LSD

We have seen thus far that N/OFQ can both presynaptically inhibit glutamate release from SF-1 neurons that contact POMC neurons and postsynaptically activate GIRK channels in POMC neurons. Coupled with the fact that N/OFQ can also activate GIRK channels in leptin receptor-bearing VMN neurons and that SF-1 neurons express leptin receptors [16, 29, 43], we then wanted to examine the postsynaptic effects of N/OFQ on SF-1 neurons directly. These recordings were taken from YFP-labeled SF-1 neurons located in the dorsomedial VMN from NR5A1-Cre male and OVX female mice that had been injected as previously mentioned. A representative example of one of these visualized recorded neurons is shown below in Fig. 12a–g. Upon application of N/OFQ, SF-1 neurons from chow-fed male mice exhibited a robust outward current as well as increase in conductance (Fig. 12h, j, k). This response was also accompanied with a robust reversible hyperpolarization as well as a decrease in firing (Fig. 12i, l, m: Student’s *t* test, *t* = 3.08158, *p* < 0.030). This postsynaptic response was very similar to the one observed in POMC neurons in that it was nearly two times larger than in slices from OVX females (Figs. 12h, k, 13a, e: multi-factorial ANOVA/LSD: *F*_E2_ = 17.63 (df = 1, *p* < 0.0005), *F*_diet_ = 0.04 (df = 1, *p* < 0.900), *F*_voltage_ = 0.14 (df = 1 *p* < 0.800), *F*_E2/diet_ = 1.97 (df = 1 *p* < 0.020), *F*_E2/ voltage_ = 0.02 (df = 1, *p* < 0.900), *F*_diet/voltage_ = 0.02 (df = 1, *p* < 0.900), *F*_E2/diet/voltage_ = 0.12 (df = 1, *p* < 0.800); Fig. 13f: multi-factorial ANOVA/LSD: *F*_E2_ = 5.63 (df = 1, *p* < 0.030), *F*_diet_ = 01.87 (df = 1, *p* < 0.200), *F*_interaction_ = 1.94 (df = 1, *p* < 0.200)), and abrogated in the presence of E_2_ (Fig. 13b, c); with no further increase in response amplitude observed in HFD-fed males (not shown). This indicates that N/OFQ modulates the activation of GIRK channels on SF-1 neurons in a sex- and E_2_-dependent fashion.

### Experiment 8: Direct injection of N/OFQ into the ARC significantly increases food intake and modulates energy expenditure in a sex- and diet- dependent

**Fig. 14:**
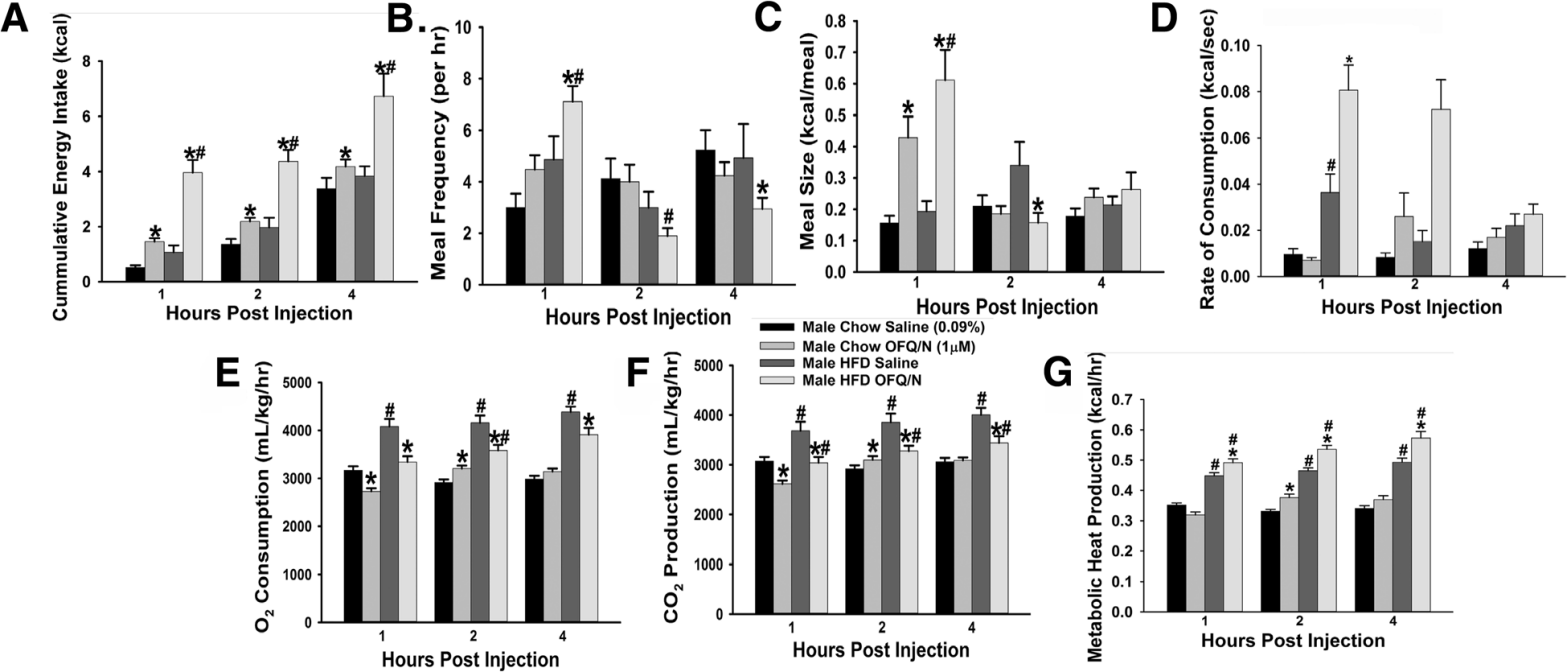
Intra-ARC N/OFQ alters energy intake and expenditure in a diet-dependent fashion. N/OFQ (0.3 nmol) significantly increased cumulative energy intake (**a**) in chow- and HFD-fed wildtype male mice compared to saline-treated controls. It also increased meal frequency (**b**), meal size (**c**), and rate of consumption (**d**) in a diet-dependent fashion. In addition, it decreased indices of energy expenditure including O_2_ consumption (**e**) and CO_2_ production (**f**) and altered metabolic heat production (**g**). Bars represent means and lines 1 S.E.M. of the cumulative food intake, meal frequency, meal size, rate of consumption, O_2_ consumption, CO_2_ production, and metabolic heat production, seen in male mice injected with either N/OFQ (0.3 nmol; *n* = 6), or its filtered 0.9% saline vehicle (0.2 μL; *n* = 6) *p < 0.05 relative to saline vehicle; repeated-measures, multi-factorial ANOVA/LSD; #*p* < 0.05 relative to chow-fed controls; repeated measures, multi-factorial ANOVA/LSD

Thus far with our in vitro studies, we have found that N/OFQ has inhibitory effects on both POMC and SF-1 neurons and decreases excitatory neurotransmission at synapses between the two. We now wanted to ascertain if these effects of N/OFQ translated into changes in energy intake and expenditure. N/OFQ significantly increased cumulative energy intake in wildtype male mice from 1 to 4 h post-injection in comparison to the saline control group, which was further potentiated by exposure to HFD (Fig. 14a: repeated measures, multi-factorial ANOVA/LSD: *F*_OFQ_ = 66.88 (df = 1, *p* < 0.0001), *F*_diet_ = 57.76 (df = 2, *p* < 0.0001), *F*_hour_ = 45.41 (df = 1, *p* < 0.0001), *F*_OFQ/diet_ = 0.17 (df = 2, *p* < 0.90), *F*_OFQ/hour_ = 18.14 (df = 1, *p* < 0.0001), *F*_diet/hour_ = 0.03 (df = 2, *p* < 1.00), *F*_OFQ/diet/hour_ = 0.12 (df = 2, *p* < 0.900); one-way ANOVA/LSD: *F*_between groups_ = 22.98 (df = 11, *p* < 0.0001)). HFD-fed, N/OFQ-treated males also had a significant increase in their meal frequency at 1 h post-injection, which reversed 2 h after administration(Fig. 14b: repeated measures, multi-factorial ANOVA/LSD: *F*_OFQ_ = 0.04 (df = 1, *p* < 0.900), *F*_diet_ = 0.02 (df = 1, *p* < 0.900), *F*_hour_ = 5.80 (df = 2, *p* < 0.004), *F*_OFQ/diet_ = 0.26 (df = 1, *p* < 0.700), *F*_OFQ/hour_ = 6.49 (df = 2, *p* < 0.005), *F*_diet/hour_ = 8.91 (df = 2, *p* < 0.005), *F*_OFQ/diet/hour_ = 0.55 (df = 2, *p* < 0.600); one-way ANOVA/LSD: *F*_between groups_ = 4.11 (df = 11, *p* < 0.0001)), while in chow animals we saw no difference. N/OFQ exerted significant increases in meal size that, again, were appreciably accentuated by the HFD (Fig. 14c: repeated measures, multi-factorial ANOVA/LSD: *F*_OFQ_ = 11.59 (df = 1, *p* < 0.0010), *F*_diet_ = 4.83 (df = 1, *p* < 0.05), *F*_hour_ = 8.13 (df = 2, *p* < 0.005), *F*_OFQ/diet_ = 0.02 (df = 1, *p* < 0.900), *F*_OFQ/hour_ = 20.51 (df = 2, *p* < 0.0001), *F*_diet/hour_ = 0.66 (df = 2, *p* < 0.60), *F*_OFQ/diet/hour_ = 2.28 (df = 2, *p* < 0.200); one-way ANOVA/LSD: *F*_between groups_ = 7.69 (df = 11, *p* < 0.0001)). As with meal frequency, N/OFQ produced a sizable increase in the rate of consumption in HFD-fed males out to 2 h after administration, with no effect seen in chow-fed animals (Fig. 14d: repeated measures, multi-factorial ANOVA/LSD: *F*_OFQ_ = 15.71 (df = 1, *p* < 0.0005), *F*_diet_ = 45.94 (df = 1, *p* < 0.0001), *F*_hour_ = 5.03 (df = 2, *p* < 0.0080), *F*_OFQ/diet_ = 6.36 (df = 1, *p* < 0.0200), *F*_OFQ/hour_ = 4.38 (df = 2, *p* < 0.020), *F*_diet/hour_ = 9.30 (df = 2, *p* < 0.0005), *F*_OFQ/diet/hour_ = 1.69 (df = 2, *p* < 0.200); one-way ANOVA/LSD: *F*_between groups_ = 10.39 (df = 11, *p* < 0.0001)). Regarding energy expenditure, N/OFQ significantly decreased O_2_ consumption for both dietary conditions at 1 h post-injection, which rebounded and significantly increased at 2 h post-injection. For the HFD-fed animals, the N/OFQ-induced decrease in O_2_ consumption was further extended until at least 4 h after administration (Fig. 14e: repeated measures, multi-factorial ANOVA/LSD: *F*_OFQ_ = 25.88 (df = 1, *p* < 0.0001), *F*_diet_ = 229.54 (df = 1, *p* < 0.0001), *F*_hour_ = 7.37 (df = 2, *p* < 0.0010), *F*_OFQ/diet_ = 26.18 (df = 1, *p* < 0.0001), *F*_OFQ/hour_ = 6.32 (df = 2, *p* < 0.005), *F*_diet/hour_ = 2.98 (df = 2, *p* < 0.06), *F*_OFQ/diet/hour_ = 1.99 (df = 2, *p* < 0.200); one-way ANOVA/LSD: *F*_between groups_ = 27.18 (df = 11, *p* < 0.0001)). A virtually identical pattern was observed in terms of the effects of N/OFQ on CO_2_ production (Fig. 14f: repeated-measures, multi-factorial ANOVA/LSD: *F*_OFQ_ = 30.73 (df = 1, *p* < 0.0001), *F*_diet_ = 7.93 (df = 1, *p* < 0.0010, *F*_hour_ = 88.30 (df = 2, *p* < 0.0001), *F*_OFQ/diet_ = 3.08 (df = 1, *p* < 0.050), *F*_OFQ/hour_ = 17.65, (df = 2, *p* < 0.0001), *F*_diet/hour_ = 0.38 (df = 2, *p* < 0.7000), *F*_OFQ/diet/hour_ = 1.94 (df = 2, *p* < 0.2000); one-way ANOVA/LSD: *F*_between groups_ = 13.78 (df = 11, *p* < 0.0001)). N/OFQ also caused an increase in metabolic heat production in chow-fed males 2 h after injection, which was more prolonged and of greater magnitude in HFD-fed males (Fig. 14g: repeated measures, multi-factorial ANOVA/LSD, *F*_OFQ_ = 8.27 (df = 1, *p* < 0.0050), *F*_diet_ = 225.69 (df = 1, *p* < 0.0001), *F*_hour_ = 5.81 (df = 2, *p* < 0.0040), *F*_OFQ/diet_ = 3.00 (df = 1, *p* < 0.090), *F*_OFQ/hour_ = 3.01 (df = 2, *p* < 0.060), *F*_diet/hour_ = 7.58 (df = 2, *p* < 0.0010), *F*_OFQ/diet/hour_ = 0.41 (df = 2, *p* < 0.7000); one-way ANOVA/LSD: F_between groups_ = 24.74 (df = 11, *p* < 0.0001)). Thus, the multi-faceted inhibitory actions of N/OFQ at anorexigenic VMN SF-1/ARC POMC synapses effectively leads to increases in energy intake and changes in energy expenditure which are further potentiated by long-term exposure to HFD.

**Fig. 15:**
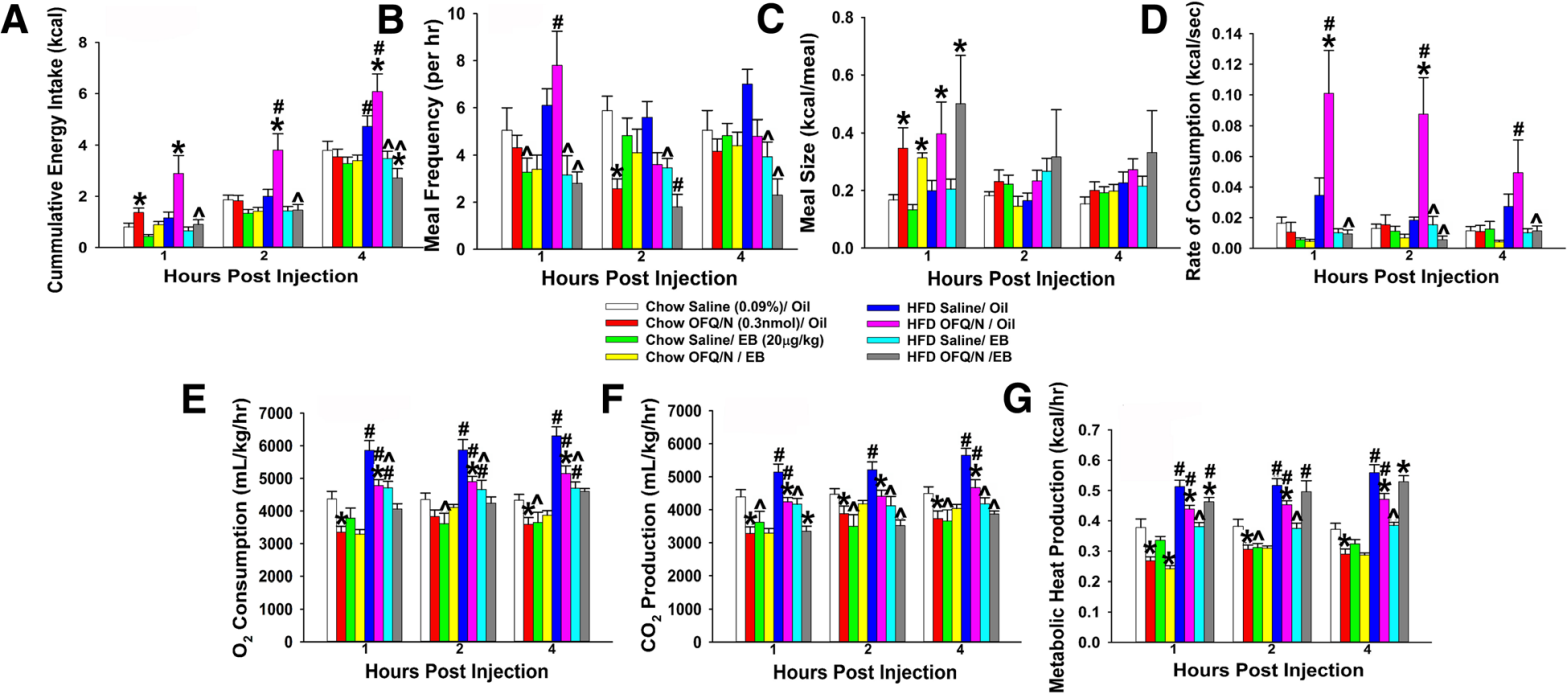
Intra-ARC N/OFQ alters energy intake and expenditure in a diet- and EB-dependent fashion. N/OFQ (0.3 nmol) significantly increased cumulative energy intake (**a**) in chow-fed wildtype, OVX female mice, which was further potentiated in HFD-fed animals compared to saline-treated controls. It also increased meal frequency (**b**), meal size (**c**) and rate of consumption (**d**) , and for **b** and **d**, these effects were significantly influenced in a diet- and EB-dependent fashion. In addition, it decreased indices of energy expenditure including O_2_ consumption (**e**), CO_2_ production (**f**), and metabolic heat production (**g**), which in the case of **e** is significantly accentuated by HFD and attenuated across the board by EB. Bars represent means and lines 1 S.E.M. of the cumulative food intake, meal frequency, meal size, rate of consumption, O_2_ consumption, CO_2_ production, and metabolic heat production, seen in OVX oil- or EB-treated female mice, injected with either N/OFQ (0.3 nmol; *n* = 6), or its filtered 0.9% saline vehicle (0.2 μL; *n* = 6) *p < 0.05 relative to saline vehicle; repeated-measures, multi-factorial ANOVA/LSD; #*p* < 0.05 relative to chow-fed controls; repeated-measures, multi-factorial ANOVA/LSD; ^*p* < 0.05 relative to sesame oil vehicle; repeated-measures, multi-factorial ANOVA/LSD

In chow-fed, oil-treated OVX females, N/OFQ produced a transient increase in cumulative energy intake that was markedly potentiated in their HFD-fed counterparts. EB treatment (20 μg/kg; s.c.) completely abolished this effect (Fig. 15a: repeated measures, multi-factorial ANOVA/LSD: *F*_OFQ_ = 13.69 (df = 1, *p* < 0.0005), *F*_E2_ = 73.13 (df = 1, *p* < 0.0001), *F*_diet_ = 24.71 (df = 1, *p* < 0.0001), *F*_hour_ = 178.99 (df = 2, *p* < 0.0001), *F*_OFQ/E2_ = 12.02 (df = 1, *p* < 0.0010), *F*_OFQ/diet_ = 5.51 (df = 1, *p* < 0.020), *F*_OFQ/hour_ = 2.44 (df = 2, *p* < 0.090), *F*_E2/diet_ = 26.50 (df = 1, *p* < 0.0001), *F*_E2/hour_ = 1.37 (df = 2, *p* < 0.300), *F*_diet/hour_ = 0.35 (df = 2, *p* < 0.8000), *F*_OFQ/E2/diet_ = 15.05 (df = 1, *p* < 0.0005), *F*_OFQ/E2/hour_ = 0.00 (df = 2, *p* < 1.00), *F*_OFQ/diet/hour_ = 0.46 (df = 2, *p* < 0.7), *F*_E2/diet/hour_ = 2.20 (df = 2, *p* < 0.200), *F*_OFQ/E2/diet/hour_ = 0.45 (df = 2, *p* < 0.700); one-way ANOVA/LSD, *F*_between groups_ = 23.46 (df = 23, *p* < 0.0001)). While we cannot ascribe the increase in energy intake caused by N/OFQ to changes in meal frequency (Fig. 15b: repeated measures, multi-factorial ANOVA/LSD: *F*_OFQ_ = 22.58 (df = 1, *p* < 0.0009), *F*_E2_ = 30.45 (df = 1, *p* < 0.0001), *F*_diet_ = 0.02 (df = 1, *p* < 1.00), *F*_hour_ = 1.50 (df = 2, *p* < 0.300), *F*_OFQ/E2_ = 0.60 (df = 1, *p* < 0.50), *F*_OFQ/diet_ = 0.00 (df = 1, *p* < 1.00), *F*_OFQ/hour_ = 4.41 (df = 2, *p* < 0.020), *F*_E2/diet_ = 18.31 (df = 1, *p* < 0.0001), *F*_E2/hour_ = 3.22 (df = 2, *p* < 0.050), *F*_diet/hour_ = 2.74 (df = 2, *p* < 0.070), *F*_OFQ/E2/diet_ = 2.00 (df = 1, *p* < 0.200), *F*_OFQ/E2/hour_ = 0.99 (df = 2, *p* < 0.400), *F*_OFQ/diet/hour_ = 1.20 (df = 2, *p* < 0.40), *F*_E2/diet/hour_ = 0.09 (df = 2, *p* < 1.00), *F*_OFQ/E2/diet/hour_ = 0.60 (df = 2, *p* < 0.60); one-way ANOVA/LSD: *F*_between groups_ = 3.71 (df = 23, *p* < 0.0001)), the hyperphagic response was associated with increases in meal size (Fig. 15c: repeated measures, multi-factorial ANOVA/LSD: *F*_OFQ_ = 16.00 (df = 1, *p* < 0.0005), *F*_E2_ = 0.83 (df = 1, *p* < 0.40), *F*_diet_ = 8.46 (df = 1, *p* < 0.005), *F*_hour_ = 2.80 (df = 2, *p* < 0.070), *F*_OFQ/E2_ = 0.00 (df = 1, *p* < 1.00), *F*_OFQ/diet_ = 1.79 (df = 1, *p* < 0.200), *F*_OFQ/hour_ = 5.97 (df = 2, *p* < 0.005), *F*_E2/diet_ = 2.07 (df = 1, *p* < 0.20), *F*_E2/hour_ = 0.09 (df = 2, *p* < 1.00), *F*_diet/hour_ = 0.19 (df = 2, *p* < 0.90), *F*_OFQ/E2/diet_ = 1.20 (df = 1, *p* < 0.30), *F*_OFQ/E2/hour_ = 0.55 (df = 2, *p* < 0.60), *F*_OFQ/diet/hour_ = 0.01 (df = 2, *p* < 1.00), *F*_E2/diet/hour_ = 0.45 (df = 2, *p* < 0.70), *F*_OFQ/E2/diet/hour_ = 0.00 (df = 2, *p* < 1.00); one-way ANOVA/LSD: *F*_between groups_ = 2.11 (df = 23, *p* < 0.005)). Moreover, the augmented N/OFQ-induced increase in energy intake seen in HFD-fed, oil-treated OVX females was paralleled by marked increases in the rate of consumption that, again, were negated by EB treatment (Fig. 15d: repeated measures, multi-factorial ANOVA/LSD: *F*_OFQ_ = 5.03 (df = 1, *p* < 0.05), *F*_E2_ = 45.62 (df = 1, *p* < 0.0001), *F*_diet_ = 36.85 (df = 1, *p* < 0.0001), *F*_hour_ = 1.55 (df = 2, *p* < 0.30), *F*_OFQ/E2_ = 10.44 (df = 1, *p* < 0.005), *F*_OFQ/diet_ = 8.99 (df = 1, *p* < 0.0040), *F*_OFQ/hour_ = 0.71 (df = 2, *p* < 0.50), *F*_E2/diet_ = 28.63 (df = 1, *p* < 0.0001), *F*_E2/hour_ = 2.04 (df = 2, *p* < 0.20), *F*_diet/hour_ = 1.33 (df = 2, *p* < 0.30), *F*_OFQ/E2/diet_ = 7.88 (df = 1, *p* < 0.010), *F*_OFQ/E2/hour_ = 0.47 (df = 2, *p* < 0.70), *F*_OFQ/diet/hour_ = 0.71 (df = 2, *p* < 0.50), *F*_E2/diet/hour_ = 1.14 (df = 2, *p* < 0.50), *F*_OFQ/E2/diet/hour_ = 1.23 (df = 2, *p* < 0.50); one-way ANOVA/LSD: *F*_between groups_ = 6.24 (df = 23, *p* < 0.0001)). In terms of energy expenditure, N/OFQ reduced O_2_ consumption in chow-fed, oil-treated OVX females that was more pervasive in HFD-fed females and diminished by EB treatment (Fig. 15e: repeated measures, multi-factorial ANOVA/LSD: *F*_OFQ_ = 25.56 (df = 1, *p* < 0.0001), *F*_E2_ = 34.15 (df = 1, *p* < 0.0001), *F*_diet_ = 116.27 (df = 1, *p* < 0.0001), *F*_hour_ = 1.93 (df = 2, *p* < 0.200), *F*_OFQ/E2_ = 12.80 (df = 1, *p* < 0.0005), *F*_OFQ/diet_ = 3.32 (df = 1, *p* < 0.10), *F*_OFQ/hour_ = 1.74 (df = 2, *p* < 0.20), *F*_E2/diet_ = 11.86 (df = 1, *p* < 0.0010), *F*_E2/hour_ = 0.02 (df = 2, *p* < 1.00), *F*_diet/hour_ = 1.10 (df = 2, *p* < 0.40), *F*_OFQ/E2/diet_ = 0.13 (df = 1, *p* < 0.800), *F*_OFQ/E2/hour_ = 0.53 (df = 2, *p* < 0.60), *F*_OFQ/diet/hour_ = 0.62 (df = 2, *p* < 0.60), *F*_E2/diet/hour_ = 0.13 (df = 2, *p* < 0.90), *F*_OFQ/E2/diet/hour_ = 0.16 (df = 2, *p* < 0.90); one-way ANOVA/LSD: *F*_between groups_ = 12.73 (df = 23, *p* < 0.0001)). N/OFQ also decreased CO_2_ production in chow- and HFD-fed, vehicle-treated OVX females that was attenuated by EB treatment (Fig. 15f: repeated measures, multi-factorial ANOVA/LSD: *F*_OFQ_ = 26.56 (df = 1, *p* < 0.0001), *F*_E2_ = 45.53 (df = 1, *p* < 0.0001), *F*_diet_ = 25.12 (df = 1, *p* < 0.0001), *F*_hour_ = 4.26 (df = 2, *p* < 0.020), *F*_OFQ/E2_ = 11.96 (df = 1, *p* < 0.0010), *F*_OFQ/diet_ = 5.04 (df = 1, *p* < 0.030), *F*_OFQ/hour_ = 2.01 (df = 2, *p* < 0.200), *F*_E2/diet_ = 12.17 (df = 1, *p* < 0.0010), *F*_E2/hour_ = 0.02 (df = 2, *p* < 1.00), *F*_diet/hour_ = 0.92 (df = 2, *p* < 0.40), *F*_OFQ/E2/diet_ = 3.47 (df = 1, *p* < 0.70), *F*_OFQ/E2/hour_ = 0.48 (df = 2, *p* < 0.700), *F*_OFQ/diet/hour_ = 0.77 (df = 2, *p* < 0.500), *F*_E2/diet/hour_ = 0.22 (df = 2, *p* < 0.90), *F*_OFQ/E2/diet/hour_ = 0.19 (df = 2, *p* < 0.90); one-way ANOVA/LSD: *F*_between groups_ = 8.42 (df = 23, *p* < 0.0001)). Similarly, N/OFQ elicited reductions in metabolic heat production in chow- and HFD-fed, vehicle-treated OVX females that, again, was largely abrogated by EB treatment (Fig. 15g: repeated measures, multi-factorial ANOVA/LSD: *F*_OFQ_ = 1.03 (df = 1, *p* < 0.40), *F*_E2_ = 21.16 (df = 1, *p* < 0.0001), *F*_diet_ = 460.52 (df = 1, *p* < 0.0001), *F*_hour_ = 9.31 (df = 2, *p* < 0.0005), *F*_OFQ/E2_ = 0.41 (df = 1, *p* < 0.60), *F*_OFQ/diet_ = 8.83 (df = 1, *p* < 0.0005), *F*_OFQ/hour_ = 0.29 (df = 2, *p* < 0.80), *F*_E2/diet_ = 1.92 (df = 1, *p* < 0.20), *F*_E2/hour_ = 0.41 (df = 2, *p* < 0.70), *F*_diet/hour_ = 3.13 (df = 2, *p* < 0.05), *F*_OFQ/E2/diet_ = 28.58 (df = 1, *p* < 0.0001), *F*_OFQ/E2/hour_ = 0.22 (df = 2, *p* < 0.90), *F*_OFQ/diet/hour_ = 0.07 (df = 2, *p* < 1.00), *F*_E2/diet/hour_ = 0.50 (df = 2, *p* < 0.70), *F*_OFQ/E2/diet/hour_ = 0.65 (df = 2, *p* < 0.60); one-way ANOVA/LSD: *F*_between groups_ = 24.79 (df = 23, *p* < 0.0001)). Thus, N/OFQ increases energy intake and decreases energy expenditure in OVX females, which is antagonized by E_2_ and, at least in some instances, potentiated by long-term HFD exposure.

## Discussion

The results generated from this project demonstrate that N/OFQ modulates energy homeostasis via pleiotropic actions at VMN SF-1/ARC POMC synapses in a sex- and diet-dependent manner. These findings are based on the following observations: (1) N/OFQ decreases leEPSC amplitude in POMC neurons upon photostimulation of SF-1 neurons via activation of NOP receptors; (2) this effect is significantly more pronounced in males than in proestrus and diestrus females; (3) HFD further accentuates the N/OFQ-induced presynaptic inhibition of excitatory neurotransmission at VMN SF-1/ARC POMC synapses; (4) N/OFQ induces a prominent, reversible outward current in both POMC and SF-1 neurons associated with an increase in conductance that robustly hyperpolarizes and decreases firing in these cells, again via activation of NOP receptors; (5) these N/OFQ-induced postsynaptic effects are sexually differentiated, fluctuate during the estrous cycle, enhanced in POMC neurons from HFD-fed females, and markedly attenuated by E_2_; and (6) N/OFQ delivered directly into the ARC increases energy intake and decreases energy expenditure, which is further potentiated by long-term exposure to HFD in males and, to a lesser extent, in OVX females, and diminished by E_2_. Collectively, these findings validate our working hypothesis that males are more responsive than females to the multi-faceted effects of N/OFQ within the hypothalamic energy balance circuitry, which can be further accentuated under conditions of diet-induced obesity/insulin resistance in sex-specific ways.

### N/OFQ pleotropically modulates neurotransmission at VMN SF-1/ARC POMC synapses in males due to the activation of the NOP receptor

We have demonstrated that N/OFQ decreases glutamatergic input from VMN SF-1 neurons onto ARC POMC neurons due to the activation of the NOP receptor. This finding aligns with other examples of N/OFQ-induced presynaptic inhibition of glutamatergic input onto neurons located in other brain regions like the suprachiasmatic nucleus (SCN), where Gompf et al. found that bath application of N/OFQ produced a concentration-dependent inhibition of glutamatergic EPSCs [15], or in the rat lateral amygdala, where extracellular application of N/OFQ was found to again dose dependently decrease EPSC amplitude [17]. It also coincides with previous reports that N/OFQ decreases EPSC amplitude in unidentified ARC neurons [16] and reduces mEPSC frequency in ARC POMC neurons, in a manner sensitive to NOP receptor antagonism or genetic ablation [25, 41]. Most importantly, this study is the first to clearly delineate the anatomical origin of the N/OFQ-sensitive glutamatergic input that impinges on POMC neurons.

We also found that N/OFQ not only modulates the presynaptic inputs onto ARC POMC neurons emanating from VMN SF-1 neurons, but that it also activates somatodendritic NOP receptors on both SF-1 and POMC neurons to hyperpolarize and thereby inhibit these cells. Again, these data are in agreement with prior reports of similar postsynaptic responses in other brain regions such as the SCN [44]. They are also congruent with previous findings in the ARC, where N/OFQ produces a robust outward current and hyperpolarization that is associated with an increase in conductance, and abrogated by NOP receptor antagonists, genetic ablation of the NOP receptor, and GIRK channel blockers [12, 25, 41].

### The pleiotropic actions of N/OFQ at VMN SF-1/ARC POMC synapses are sexually differentiated

Our present study also demonstrates that both the pre- and postsynaptic effects of N/OFQ are sexually differentiated; with males being more sensitive than females during certain stages of their estrous cycle. This is consistent with other sex differences we have seen previously at this particular synaptic connection, with males being more sensitive to retrograde, EC-mediated inhibition of glutamatergic input emanating from SF-1 neurons onto POMC neurons [21]. N/OFQ has also proven to have gender-specific modulations in other regions. Flores et al. [33] also found that N/OFQ inhibits NMDA-evoked excitatory responses in trigeminal nociceptive neurons from male and OVX female rats, but not in proestrus or OVX, estradiol-treated female rats. In addition, Claiborne et al. reported that N/OFQ failed to produce antinociceptin in proestrus rats, and estradiol dose dependently diminished the antinociception seen in OVX females [34]. Conversely, testosterone facilitated the antinociceptive effect of N/OFQ [34]. Moreover, activation of ERα and Gq-mER attenuates NOP-mediated antinociceptin in males and OVX females [35], as well as the activation of GIRK channels in ARC POMC neurons [37], via signal transduction pathways that include extracellular signal-regulated kinase, PKC, PKA, PI3K, and neuronal nitric oxide synthase (nNOS).

In the present study, we saw that the pleiotropic actions of N/OFQ at SF-1/POMC synapses varied across the estrous cycle. For example, during metestrus, the NOP receptor-mediated activation of GIRK channels in POMC neurons is similar to that observed in males, whereas in diestrus, proestrus, and estrus females it is markedly attenuated. Moreover, the NOP receptor-mediated presynaptic inhibition of glutamatergic input from SF-1 neurons onto POMC neurons is significantly diminished during diestrus and proestrus as compared to males, whereas in estrus and metestrus females the number of functional inputs is greatly reduced. The fluctuations in the N/OFQ-induced responses are similar to what we have demonstrated previously in OVX, estradiol-primed females—with and without progesterone treatment. Indeed, Borgquist et al. found that progesterone treatment following estradiol priming restores the responsiveness of POMC neurons to the postsynaptic, N/OFQ-induced activation of GIRK channels that was blunted by estradiol treatment alone [36]. Progesterone also reinstates the ability of N/OFQ to presynaptically inhibit glutamatergic input, and markedly dampens the ability of N/OFQ to presynaptically inhibit GABAergic input, onto POMC neurons in estradiol-primed females [36]. This would explain why, during diestrus and proestrus, when estradiol levels are on the rise [45, 46], the pleiotropic actions of N/OFQ at SF-1/POMC synapses are appreciably diminished. Indeed, hypothalamic circuits are most sensitive to the feedback actions of estradiol during this period [47, 48]. Conversely, while progesterone has been reported to peak during estrus [45, 49], it has also been documented to be elevated during metestrus as well [46, 50], and it is clear that the progesterone-induced increase in the responsiveness of SF-1/POMC synapses to N/OFQ manifests during this stage of the cycle.

### Diet-induced obesity further modifies N/OFQ’s pleotropic effects at VMN SF-1/ARC POMC synapses in a sexually disparate manner

This present study also demonstrates that long-term exposure to HFD, in males, further accentuates the N/OFQ-induced decrease in excitatory neurotransmission at this specific synapse. Diet-induced obesity has long been associated with dysregulated neuroendocrine function within the hypothalamic energy balance circuitry. For instance, Fabelo et al. found previously that males exposed long-term to HFD exhibit a more pronounced reduction in leEPSC amplitude in POMC neurons upon optogenetic stimulation of SF-1 neurons that occurs via enhanced retrograde EC-mediated signaling, whereas in females this is due to a loss of functional excitatory synapses altogether [21]. The alterations caused by diet-induced obesity/insulin resistance are associated with disordered PI3K/Akt signaling in the ARC and VMN. For example, the sexually differentiated increase in inhibitory EC tone at SF-1/POMC synapses seen with diet-induced obesity/insulin resistance is linked to reduced PI3K/Akt signaling in male but not female animals [21, 38]. On the other hand, diet-induced obesity promotes an insulin-dependent increase in PI3K signaling in the VMN [51]. PI3K and the energy sensor AMPK are counter-regulatory signaling molecules involved in the hypothalamic control of energy balance [52, 53]. Thus, the reduction in ARC PI3K/Akt signaling seen with obese/insulin resistance could pave the way for testosterone-induced AMPK activation in males that increases EC and N/OFQ tone at SF-1/POMC synapses [20, 54]. Interestingly, diet-induced obesity/insulin resistance enhances NOP receptor-mediated activation of GIRK channels in POMC neurons from female but not male animals. Coupled with the accentuated EC- and N/OFQ-induced presynaptic inhibition of glutamatergic input from SF-1 neurons onto POMC neurons, it stands to reason that the enhancement of this G_i/o_-coupled metabotropic receptor-mediated response may represent a more global form of adaptive plasticity occurring with diet-induced obesity/insulin resistance at these synapses (see Fig. 15).

We also found that E_2_ decouples NOP receptors from their GIRK channels in both POMC and SF-1 neurons from both chow-fed and obese females. This is entirely consistent with prior reports demonstrating that in POMC neurons, or in excitatory inputs impinging upon POMC neurons, E_2_ rapidly uncouples metabotropic G_i/o_-coupled receptors like μ-opioid, GABA_B_, and CB1 receptors from their effector systems [32, 55–57]. This occurs via activation of ERα and the G_q_-coupled mER via signaling pathways that include PLC, PI3K, nNOS, PKC, and PKA [32, 37, 57, 58]. In addition, the decoupling of inhibitory ORL1 receptors from GIRK channels in SF-1 neurons lies in agreement with the ERα/PI3K-mediated increase in the excitability of these cells [59]. Moreover, knockout of ERα in VMN SF-1 neurons leads to reduced energy expenditure, whereas in POMC neurons it leads to hyperphagia [60]. Given that both SF-1 and POMC neurons are anatomical substrates for the actions of insulin [38, 51, 61], this ability of E_2_ to markedly attenuate inhibitory NOP receptor-mediated neurotransmission at every node comprising these anorexigenic VMN SF-1/ARC POMC synapses may represent a novel means of protection against the development of central insulin resistance.

### Direct administration of N/OFQ into the ARC causes a time-dependent increase food intake and decrease in energy expenditure

Consistent with the ability of N/OFQ to inhibit both SF-1 and POMC neurons, as well glutamate release at the synapses formed between them, our study found that direct administration of N/OFQ into the ARC causes an increase in energy intake corresponding with parallel changes in meal pattern, as well as a decrease in energy expenditure. These effects were prominent in male and OVX female animals and markedly diminished by E_2_. We also found that the N/OFQ-induced increases in energy intake and various indices of meal pattern are more pronounced in HFD-fed males and OVX females, but not in OVX, EB-treated females. The N/OFQ-induced changes in energy intake that we observed presently are congruent with the findings of Matushita et al., who reported that the N/OFQ-induced hyperphagia and increased adiposity were further exaggerated in animals exposed to a HFD [26]. The ability of E_2_ to antagonize these effects is in keeping with numerous prior demonstrations that it negatively modulates the signal transduction elicited by orexigenic, G_i/o_-coupled receptors (for review see [32, 62]). Polidori et al. examined the site-specific effect of N/OFQ in a number of different limbic and hypothalamic structures and found that the ARC is by far the region most sensitive to the hyperphagic effects of the neuropeptide [27]. While Matsushita et al. found no change in rectal temperature or spontaneous locomotor activity with N/OFQ [26], NOP receptor knockout mice and NOP antisense-treated rats are reported to exhibit higher core body temperatures than their respective controls [5, 63]. This latter finding is indicative of a decrease in energy expenditure, which aligns with our findings that N/OFQ decreased O_2_ consumption and CO_2_ production, as well as metabolic heat production in the OVX female. These actions are appreciably attenuated by E_2_ in both chow- and HFD-fed OVX females and further potentiated in obese males.

## Conclusion

**Fig. 16:**
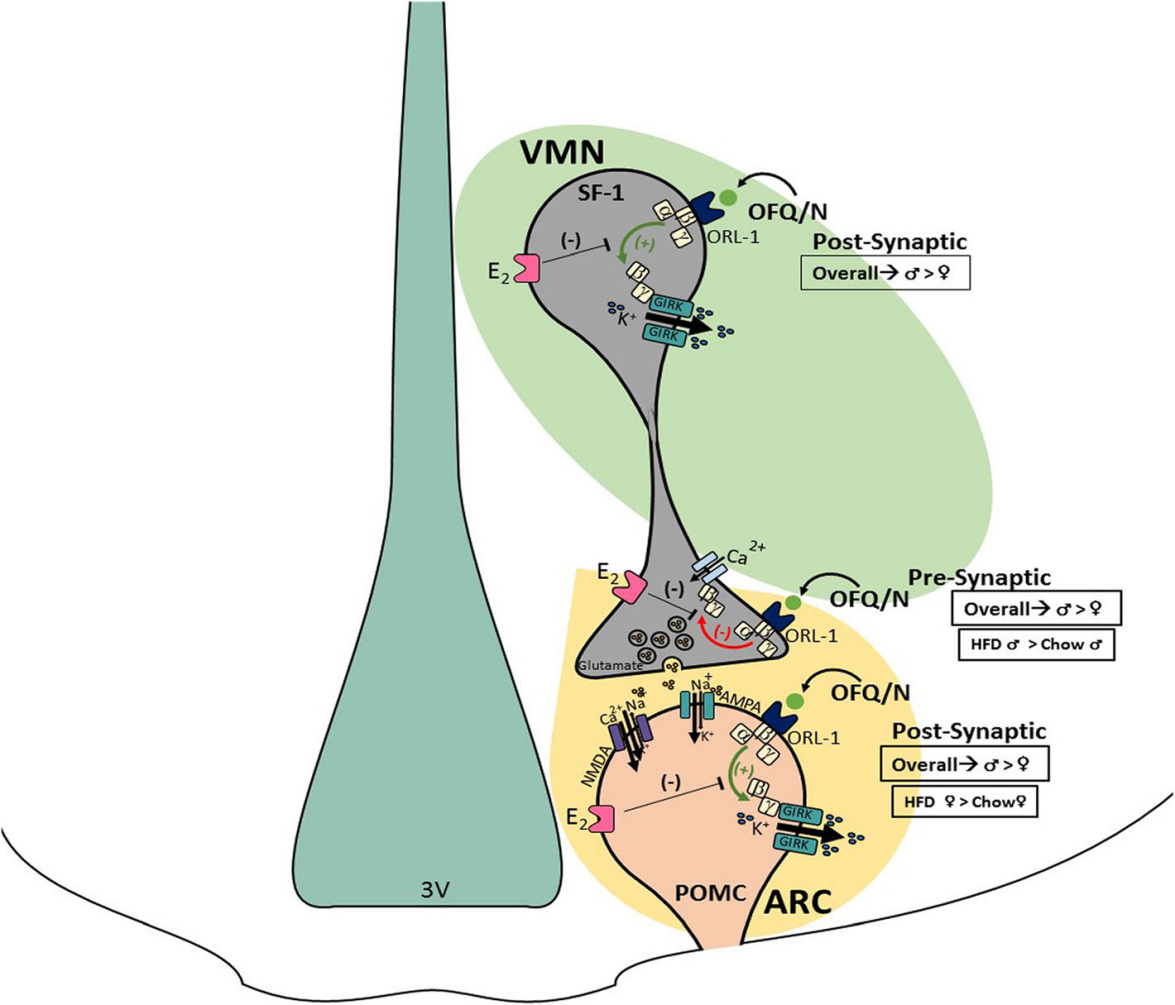
Schematic illustrating the connectivity and pleiotropic NOP receptor signaling occurring at VMN SF-1/ARC POMC synapses. N/OFQ activates somatodendritic NOP receptors that activate GIRK channels in SF-1 neurons within the dorsomedial VMN and in POMC neurons within the ARC. This leads to membrane hyperpolarization and a cessation of firing in these two neuronal populations. OFQ can also activate NOP receptors on SF-1 nerve terminals to presynaptically inhibit glutamate release onto ARC POMC neurons. The pleiotropic actions of N/OFQ at VMN SF-1/ARC POMC synapses are greater in males than in females. Moreover, the N/OFQ-induced decrease in glutamate release at SF-1/POMC synapses is accentuated in obese males but not females, whereas the postsynaptic activation of GIRK channels in POMC neurons is heightened in obese females but not males. Regardless of dietary status, these multi-faceted, NOP receptor-mediated effects are attenuated by E_2_

In conclusion, the results generated during this project provide compelling support for the idea that N/OFQ modulates energy homeostasis via inhibition of excitatory neurotransmission at VMN SF-1/ARC POMC synapses. It does this by presynaptically decreasing glutamate release into the synaptic cleft and by activating GIRK channels in both cell types, via activation of the NOP receptor in a sex- and diet-dependent manner (Fig. 16). Our findings verify our working hypothesis that males are more responsive than females to the pleiotropic actions of N/OFQ, which can be further accentuated by diet-induced obesity in a sex-specific fashion. These data have important mechanistic and therapeutic ramifications for the treatment of conditions ranging from cachexia to obesity/insulin resistance to food addiction.

## Additional files

Additional file 1: **Figure S1.** Post hoc identification of POMC neurons from NR5A1-Cre mice after visualized optogenetic whole cell patch clamp recording. **A**, ChR2 labeling in the VMN of a male NR5A1-Cre mouse visualized at × 4 with enhanced yellow fluorescent protein (YFP) 2 weeks after injection with a ChR2-containing virus. Of note is the gradient extending from the dorsomedial VMN (VMHDM) to the ventrolateral VMN (VMHVL) that is characteristic of the distribution of VMN SF-1 neurons. **B**, Labeling of ChR2-containing fibers in the ARC visualized with YFP. **C**, An infrared, differential interference contrast (DIC) image taken of an ARC neuron in close proximity to the YFP-labeled fibers seen in B. **D**, Biocytin labeling of the cell in A visualized with streptavidin/Alexa Fluor 546. **E**, An antibody directed against cocaine- and amphetamine-regulated transcript (CART), a phenotypic marker of POMC neurons, immunolabels the cell in C as visualized with Alexa Fluor 488. **F**, A composite overlay of the biocytin/CART labeling seen in the cell in A. Unless otherwise indicated, all photomicrographs were taken at × 40. The patch electrode representations outlined by dashed lines in (D–F) indicate that the images were captured after processing for immunohistofluorescence. (JPG 9418 kb)

Additional file 2: **Figure S2.** Visualized patch recording conducted in immunohistochemically identified eGFP-POMC neurons. **A**, GFP labeling of ARC neurons at × 4 magnification. **B**, GFP labeling of the recorded ARC neuron at × 40 magnification just prior to releasing positive pressure and acquisition of a GΩ seal. The dashed lines represent the outline of the patch pipette. **C**, Infrared direct interference contrast (DIC) image of the same neuron. **D**, Biocytin labeling of the cell in C (indicated with dashed arrow) visualized with streptavidin/AF546. **E**, GFP labeling of the same cell seen in B, C and D. Surrounding eGFP-filled neurons are indicated by solid arrows. **F**, An antibody directed against a-MSH immunolabels the cell in (**C**) as visualized with AF350. **G**, a composite overlay of the biocytin/GFP/a-MSH labeling seen in the cell. Panels D–G were photographed at × 20. The calibration bar equals 10 μm. (JPG 7753 kb)

Additional file 3: **Figure S3.** N/OFQ robustly hyperpolarizes POMC neurons in male guinea pigs. **A**, Representative membrane voltage trace that shows the reversible N/OFQ-induced hyperpolarization and electrical silencing. **B**, Composite bar graph that illustrates the extent of the hyperpolarization and suppression of neuronal firing (*n* = 5). Bars represent means and vertical lines 1 SEM. **C**, Biocytin labeling (visualized with streptavidin/AF546) of the cell from which the recording seen in A was taken. **D**, The α-MSH labeling (visualized with AF488) of the same cell. **E**, Composite overlay. *p < 0.05, Student’s *t* test. (JPG 6238 kb)

Additional file 4: **Figure S4.** The N/OFQ-induced activation of GIRK channels in guinea pig POMC neurons is also sexually differentiated. Membrane current traces show the N/OFQ-induced outward current in intact male guinea pigs (A; *n* = 6) and the blunted effect seen in the periovulatory female guinea pig (B; *n* = 3). The composite bar graph (**C**) further illustrate the comparatively robust N/OFQ-induced increase in the slope conductance in male guinea pigs relative to female guinea pigs at this particular stage of the cycle. Bars represent means while vertical lines indicate 1 SEM. *p < 0.05, multi-factorial ANOVA/LSD). (JPG 5318 kb)

## Abbreviations

ARC: Arcuate nucleus
EB: Estradiol benzoate
EPSCs: Excitatory postsynaptic currents
ER: Estrogen receptor
GFP: Green fluorescent protein
GIRK: G protein-gated inwardly rectifying K^+^
GPCR: G protein coupled receptor
HFD: High-fat diet
leEPSC: Light-evoked excitatory postsynaptic current
N/OFQ: Nociceptin/orphanin FQ
NOP: Nociceptin opioid peptide
OVX: Ovariectomized
POMC: Proopiomelanocortin
SF: Steroidgenic factor
VMN: Ventromedial nucleus
YFP: Yellow fluorescent protein
