# Supplementary figures and images for: Nociceptin/orphanin FQ modulates energy homeostasis through inhibition of neurotransmission at VMN SF-1/ARC POMC synapses in a sex- and diet-dependent manner
Source: Biol Sex Differ. 2019 Feb 12;10:9. doi: 10.1186/s13293-019-0220-3 (PMC6373052; doi:10.1186/s13293-019-0220-3)

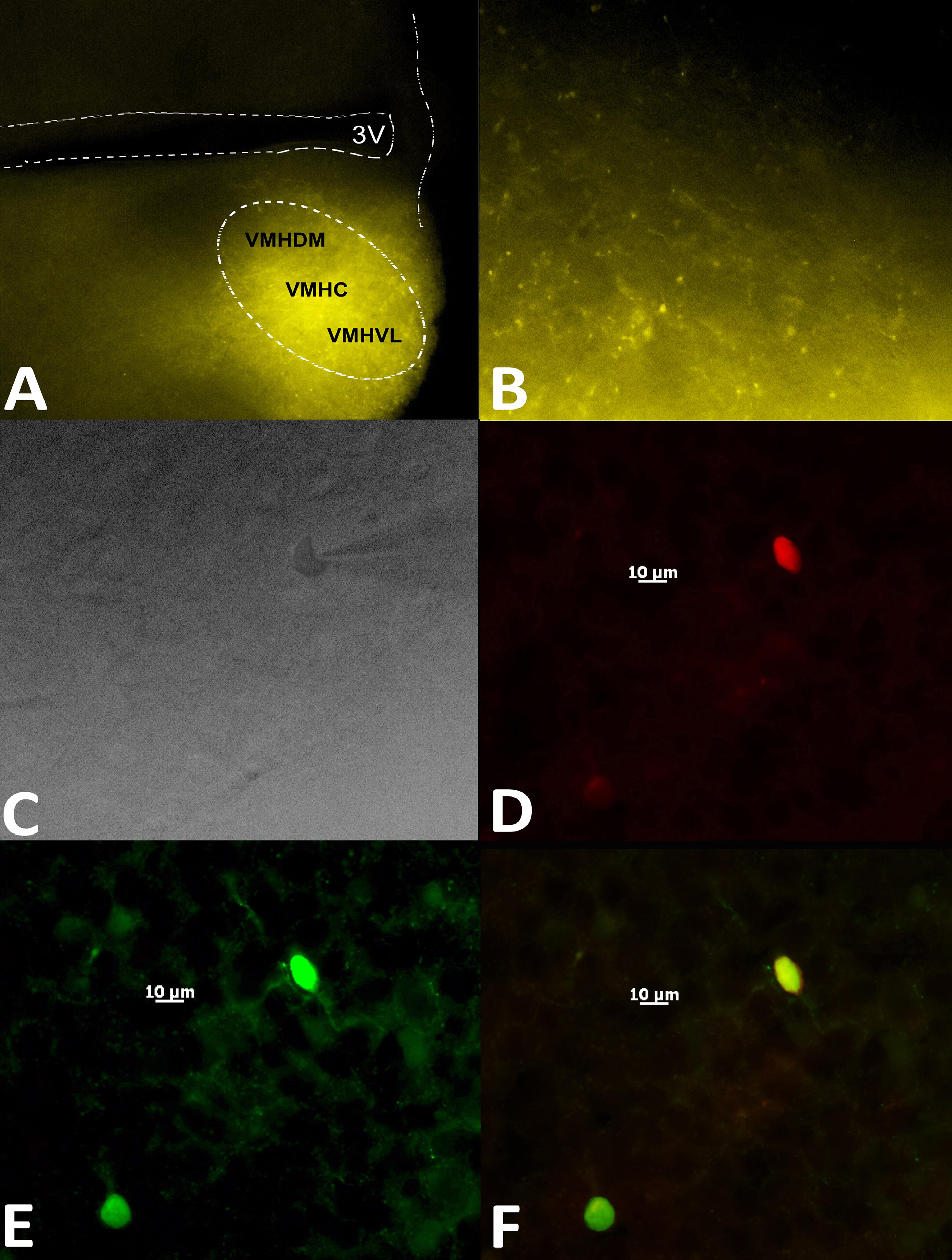

Supplement: Supplementary file 1 — Figure S1. Post hoc identification of POMC neurons from NR5A1-Cre mice after visualized optogenetic whole cell patch clamp recording. A, ChR2 labeling in the VMN of a male NR5A1-Cre mouse visualized at × 4 with enhanced yellow fluorescent protein (YFP) 2 weeks after injection with a ChR2-containing virus. Of note is the gradient extending from the dorsomedial VMN (VMHDM) to the ventrolateral VMN (VMHVL) that is characteristic of the distribution of VMN SF-1 neurons. B, Labeling of ChR2-containing fibers in the ARC visualized with YFP. C, An infrared, differential interference contrast (DIC) image taken of an ARC neuron in close proximity to the YFP-labeled fibers seen in B. D, Biocytin labeling of the cell in A visualized with streptavidin/Alexa Fluor 546. E, An antibody directed against cocaine- and amphetamine-regulated transcript (CART), a phenotypic marker of POMC neurons, immunolabels the cell in C as visualized with Alexa Fluor 488. F, A composite overlay of the biocytin/CART labeling seen in the cell in A. Unless otherwise indicated, all photomicrographs were taken at × 40. The patch electrode representations outlined by dashed lines in (D–F) indicate that the images were captured after processing for immunohistofluorescence. (JPG 9418 kb) [file 13293_2019_220_MOESM1_ESM.jpg]

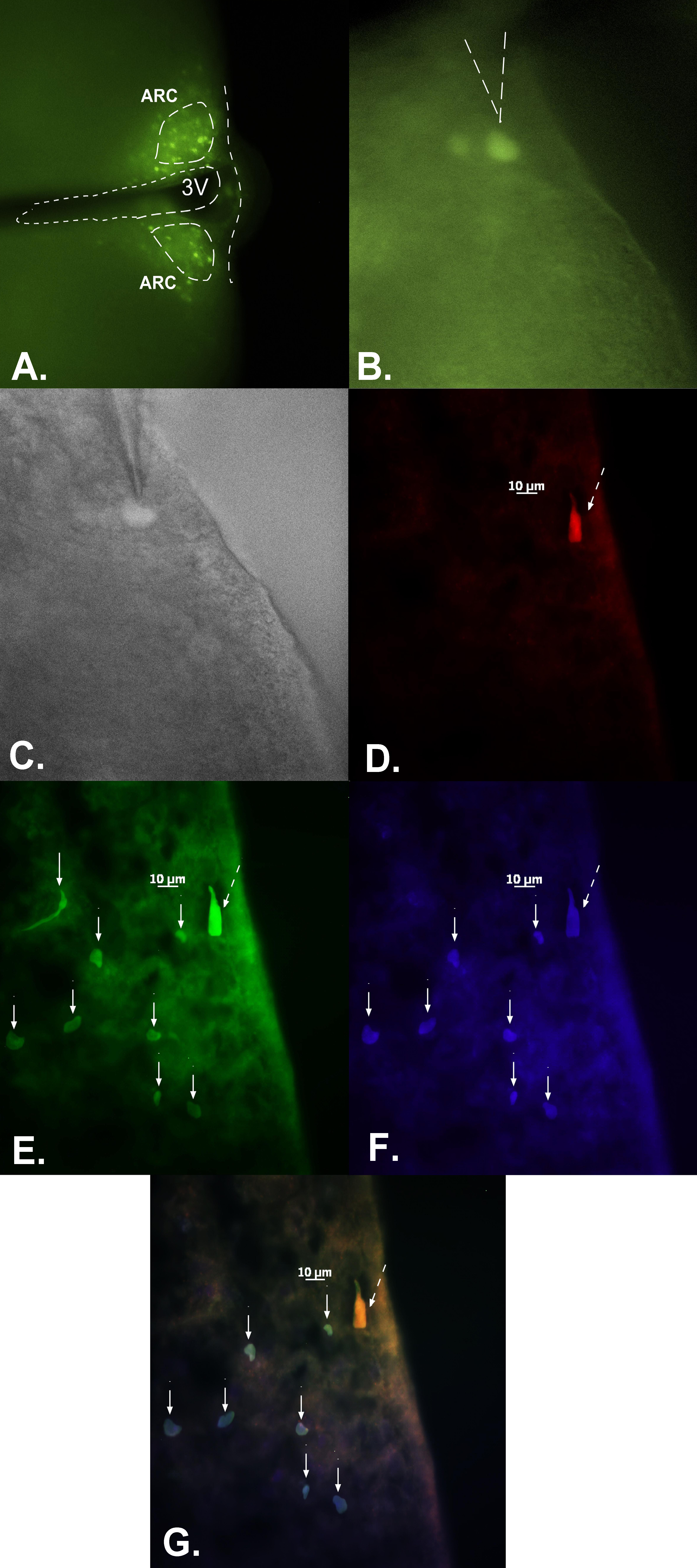

Supplement: Supplementary file 2 — Figure S2. Visualized patch recording conducted in immunohistochemically identified eGFP-POMC neurons. A, GFP labeling of ARC neurons at × 4 magnification. B, GFP labeling of the recorded ARC neuron at × 40 magnification just prior to releasing positive pressure and acquisition of a GΩ seal. The dashed lines represent the outline of the patch pipette. C, Infrared direct interference contrast (DIC) image of the same neuron. D, Biocytin labeling of the cell in C (indicated with dashed arrow) visualized with streptavidin/AF546. E, GFP labeling of the same cell seen in B, C and D. Surrounding eGFP-filled neurons are indicated by solid arrows. F, An antibody directed against a-MSH immunolabels the cell in (C) as visualized with AF350. G, a composite overlay of the biocytin/GFP/a-MSH labeling seen in the cell. Panels D–G were photographed at × 20. The calibration bar equals 10 μm. (JPG 7753 kb) [file 13293_2019_220_MOESM2_ESM.jpg]

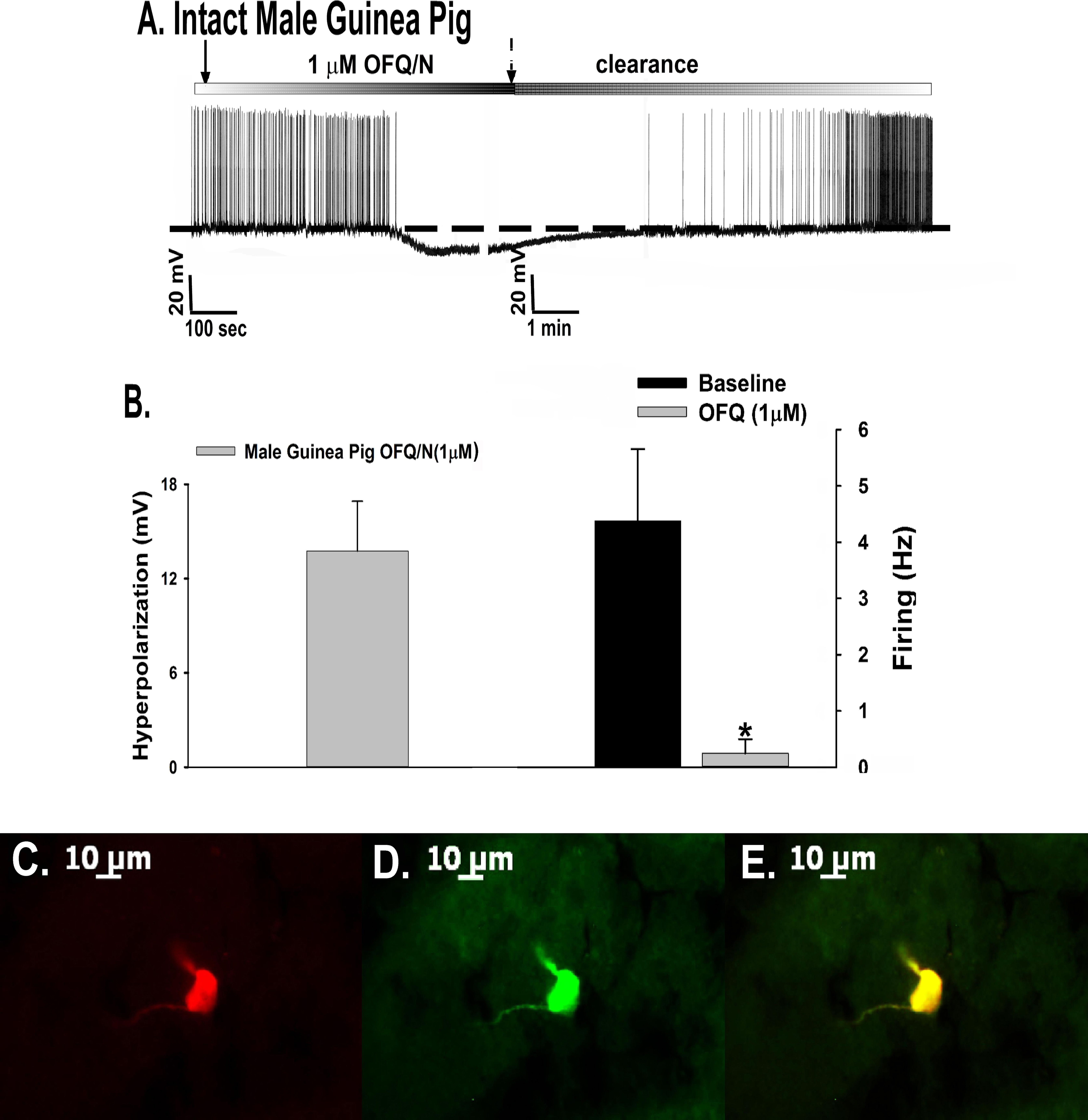

Supplement: Supplementary file 3 — Figure S3. N/OFQ robustly hyperpolarizes POMC neurons in male guinea pigs. A, Representative membrane voltage trace that shows the reversible N/OFQ-induced hyperpolarization and electrical silencing. B, Composite bar graph that illustrates the extent of the hyperpolarization and suppression of neuronal firing (n = 5). Bars represent means and vertical lines 1 SEM. C, Biocytin labeling (visualized with streptavidin/AF546) of the cell from which the recording seen in A was taken. D, The α-MSH labeling (visualized with AF488) of the same cell. E, Composite overlay. *p < 0.05, Student’s t test. (JPG 6238 kb) [file 13293_2019_220_MOESM3_ESM.jpg]

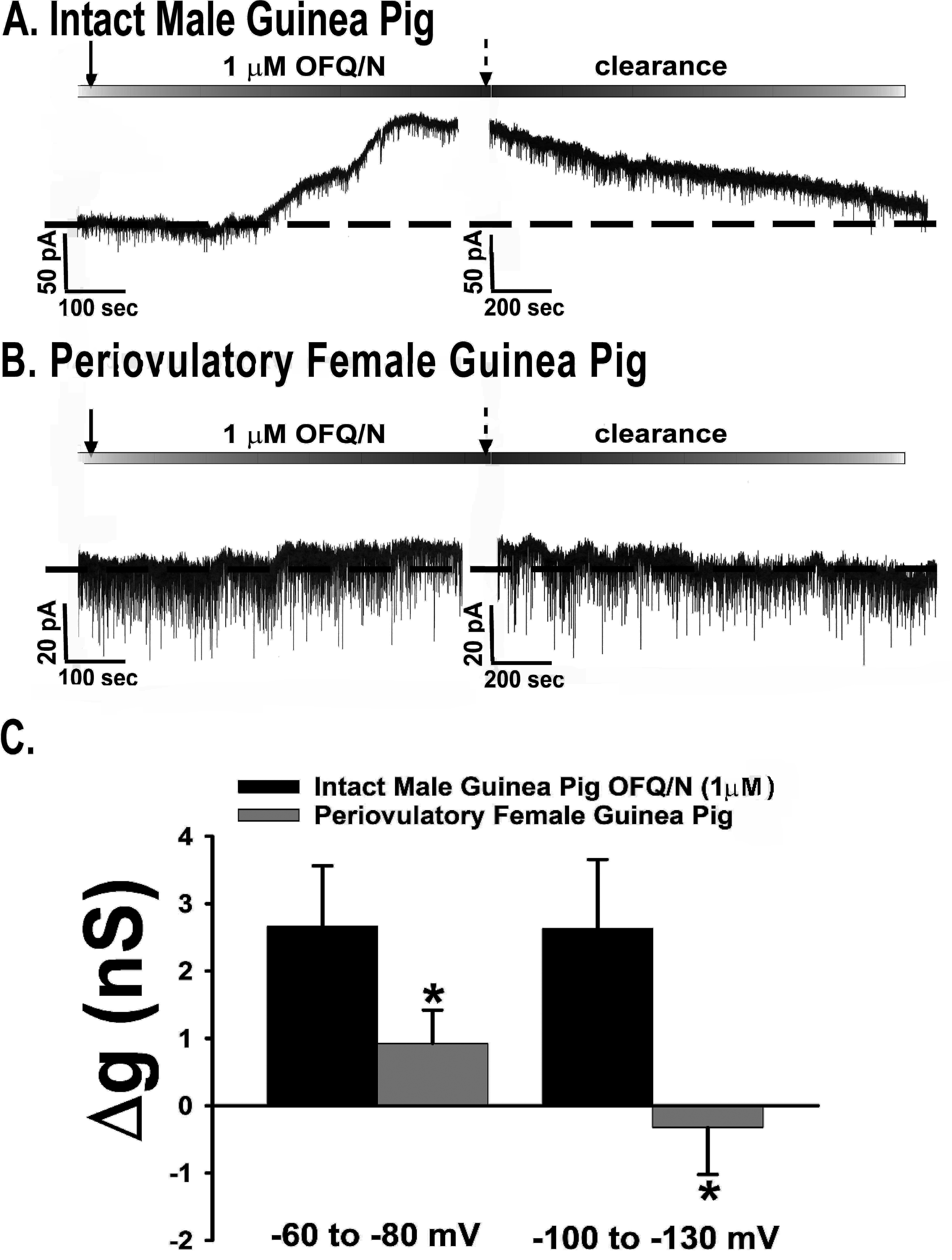

Supplement: Supplementary file 4 — Figure S4. The N/OFQ-induced activation of GIRK channels in guinea pig POMC neurons is also sexually differentiated. Membrane current traces show the N/OFQ-induced outward current in intact male guinea pigs (A; n = 6) and the blunted effect seen in the periovulatory female guinea pig (B; n = 3). The composite bar graph (C) further illustrate the comparatively robust N/OFQ-induced increase in the slope conductance in male guinea pigs relative to female guinea pigs at this particular stage of the cycle. Bars represent means while vertical lines indicate 1 SEM. *p < 0.05, multi-factorial ANOVA/LSD). (JPG 5318 kb) [file 13293_2019_220_MOESM4_ESM.jpg]
